# Single-Cell Omics for Transcriptome CHaracterization (SCOTCH): isoform-level characterization of gene expression through long-read single-cell RNA sequencing

**DOI:** 10.1101/2024.04.29.590597

**Published:** 2024-04-30

**Authors:** Zhuoran Xu, Hui-Qi Qu, Joe Chan, Charlly Kao, Hakon Hakonarson, Kai Wang

**Affiliations:** 1. Graduate Group in Genomics and Computational Biology, University of Pennsylvania Perelman School of Medicine, Philadelphia, PA, 19104, USA; 2. The Center for Applied Genomics, Children’s Hospital of Philadelphia, Philadelphia, Pennsylvania, 19104, USA.; 3. Department of Pediatrics, The Perelman School of Medicine, University of Pennsylvania, Philadelphia, Pennsylvania, 19104, USA.; 4. Raymond G. Perelman Center for Cellular and Molecular Therapeutics, Children’s Hospital of Philadelphia, Philadelphia, PA, 19104, USA; 5. Department of Pathology and Laboratory Medicine, University of Pennsylvania, Philadelphia, PA, 19104, USA

## Abstract

The advent of long-read single-cell transcriptome sequencing (lr-scRNA-Seq) represents a significant leap forward in single-cell genomics. With the recent introduction of R10 flowcells by Oxford Nanopore, we propose that previous computational methods designed to handle high sequencing error rates are no longer relevant, and that the prevailing approach using short reads to compile “barcode space” (candidate barcode list) to de-multiplex long reads are no longer necessary. Instead, computational methods should now shift focus on harnessing the unique benefits of long reads to analyze transcriptome complexity. In this context, we introduce a comprehensive suite of computational methods named Single-Cell Omics for Transcriptome CHaracterization (SCOTCH). Our method is compatible with the single-cell library preparation platform from both 10X Genomics and Parse Biosciences, facilitating the analysis of special cell populations, such as neurons, hepatocytes and developing cardiomyocytes. We specifically re-formulated the transcript mapping problem with a compatibility matrix and addressed the multiple-mapping issue using probabilistic inference, which allows the discovery of novel isoforms as well as the detection of differential isoform usage between cell populations. We evaluated SCOTCH through analysis of real data across different combinations of single-cell libraries and sequencing technologies (10X + Illumina, Parse + Illumina, 10X + Nanopore_R9, 10X + Nanopore_R10, Parse + Nanopore_R10), and showed its ability to infer novel biological insights on cell type-specific isoform expression. These datasets enhance the availability of publicly available data for continued development of computational approaches. In summary, SCOTCH allows extraction of more biological insights from the new advancements in single-cell library construction and sequencing technologies, facilitating the examination of transcriptome complexity at the single-cell level.

## Introduction

The development of single-cell RNA sequencing (scRNA-Seq) technology represents a transformative force in genomics, enhancing our comprehension of cellular heterogeneity and the dynamic nature of genetic expression to unparalleled levels^1–3^. However, when paired with conventional short-read sequencing methods, scRNA-seq encounters limitations in fully capturing complex genomic regions and providing comprehensive view of genetic variants and transcriptomic diversity^4, 5^. These limitations become particularly pronounced in the context of detecting differential isoform splicing and discovering novel isoforms, as short reads cannot cover entire transcript lengths, leading to incomplete or ambiguous reconstructions of splice variants. In contrast, long-read sequencing technologies, such as those from PacBio and Oxford Nanopore Technologies, offer a significant advantage by spanning entire gene or transcript lengths^6–9^. Consequently, the emergence of long-read scRNA-Seq (lr-scRNA-Seq) marks a major advancement in the field. It delivers a more nuanced view of the transcriptomic landscape within individual cells, achieving contiguous reading on transcripts and allowing for isoform-level inference of gene expression. Such progress is essential for elucidating complex splicing events and transcript diversity, which are crucial for a deeper understanding of cellular function and disease mechanisms^10–13^.

In recent years, various long-read single-cell technologies have been developed, such as LR-Split-seq^14^, Nanopore-specific adaptation of 10X Genomics Chromium^15^, FLASH-Seq^16^, among others, with each offering unique advantages. For instance, Parse Bioscience employs a split-pool combinatorial barcoding strategy, which allows the simultaneous sequencing of multiple samples in each experiment. This approach is particularly beneficial for analyzing special cell populations, such as neurons, hepatocytes and developing cardiomyocytes, that cannot be readily assayed by the 10X Genomics platform unless single-nucleus RNA sequencing (snRNA-Seq) is used. At the same time, the emergence of long-read sequencing also introduces new challenges, notably the historically higher error rates associated with this technology. These error rates, associated with older generation of flowcells, complicate the process of identifying barcodes (such as cell barcodes and unique molecular identifiers (UMIs)) to de-multiplex reads and assign them to specific cells, leading to an urgent need for the development of computational tools, such as LongCell^17^ and FLAMES^18^, aimed at correcting these inaccuracies and ensuring efficient downstream data analysis. In many cases, paired short-read sequencing is performed together with long-read sequencing, to “guide” the process to correct for errors and improve barcode assignment accuracy^19–21^

More recently, the introduction of R10 flowcells with v14 chemistry by Oxford Nanopore has reduced the per-base sequencing error rates to approximately 1%^22, 23^. In principle, this dramatically reduced error rate made previous computational methods that were designed to handle high sequencing error rates less relevant nowadays. Additionally, we expect that the conventional practice of using paired short-read sequencing to compile a candidate barcode list for de-multiplexing long reads has become unnecessary. Instead, computational methods should shift their focus towards exploiting the unique advantages of long reads to analyze transcriptome complexity, such as alternative splicing, alternative promoter usage, or alternative polyadenylation sites, without relying on short reads. Although computational approaches for short-read scRNA-seq have been well developed, focusing mainly on gene count quantification and cellular population clustering, they are not directly transferable to lr-scRNA-seq data to assess isoform-specific expression levels and differential isoform usage across cell populations. Currently, LongCell is among the only tools specifically developed for accurate isoform quantification in single-cell and spatial spot barcoded long-read sequencing data, showcasing the potential of long reads in revealing complex splicing patterns and transcript diversity^17^. However, LongCell primarily focuses on correcting the high error rates of R9 flowcells and employs a statistical framework to compare exon inclusion levels between cell populations, which limits its ability to identify novel isoforms and compare isoform usage differences directly. In this context, the development of new computational methods is imperative to harness the full potential of lr-scRNA-Seq to unravel the intricacies of genomic and transcriptomic diversity at the single-cell level. The integration of advanced computational algorithms with long-read sequencing data stands to revolutionize our understanding of cellular processes, paving the way for breakthroughs in biomedical research and personalized medicine.

In the current study, we have generated a comprehensive set of benchmarking datasets employing both short-read scRNA-Seq protocols on the Illumina platform and long-read scRNA-Seq protocols on the Oxford Nanopore platform, encompassing different versions of flow cells (R9 and R10) and single cell preparation protocols (10X Genomics and Parse Biosciences) (Table 1). Our examination of technical issues involved in lr-scRNA-seq data has demonstrated that recent technological advancements have rendered some of the previous concerns no longer relevant, eliminating the need for parallel short-read sequencing. Therefore, we introduce Single-Cell Omics for Transcriptome CHaracterization (SCOTCH), a suite of computational pipeline and statistical framework tailored for lr-scRNA-seq data analysis. SCOTCH is compatible with both 10X Genomics and Parse Bioscience single-cell preparation libraries, exceling in comparing transcript usage differences between cell populations and identifying isoform switching events with the ability to identify novel isoforms. We showcase its ability to unveil novel biological insights from lr-scRNA-seq data, which offers information on the transcript-level resolution that short-read data cannot achieve. Our study suggests that lr-scRNA-seq significantly enhances our understanding of genome regulation and various biological phenomena from a single cell perspective. Furthermore, the data sets on five different technical approaches from our study enhance the availability of publicly available data for continued development of computational approaches.

## Results

### SCOTCH workflow and experimental design

**Figure 1.**
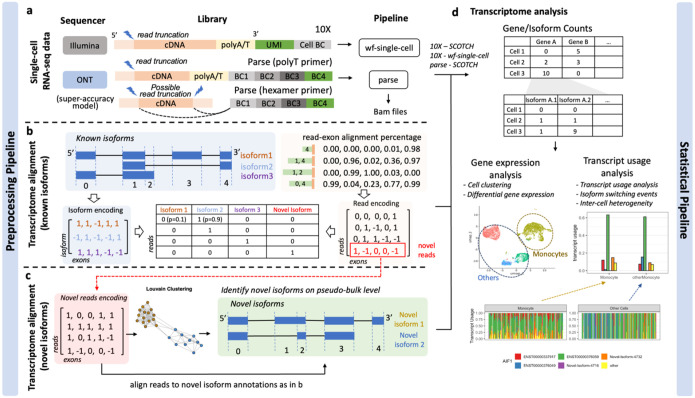
Workflow of the SCOTCH pipeline for long-read scRNA-seq analysis. (**a**) For either the 10X Genomics or Parse Biosciences single-cell library construction method, the cell barcodes and UMIs are extracted using vendor-supplied protocols. SCOTCH accounts for potential read truncations depending on primers used. (**b**) SCOTCH dissects known isoform annotations into non-overlapping exons and maps reads to each exon. Both isoform annotations and reads are encoded using presence and absence of exons. Reads are then mapped to known isoforms through a compatible matrix. (**c**) Novel reads that are unmappable to any known isoforms will be clustered to identify novel isoforms on pseudo-bulk level. Each novel read will then be aligned to novel isoform annotation using the same method as in b. (**d**) PBMC data generated using 10X Genomics or parse libraries that preprocessed by SCOTCH or wf-single-cell pipelines are used for transcriptome analysis by SCOTCH. Gene-level count matrix enables conventional gene expression analysis, whereas isoform-level count matrix facilitates transcript usage analysis.

In the current study, we introduce SCOTCH, a suite of computational pipeline and statistical framework specifically developed for analyzing lr-scRNA-seq data, as depicted in Figure 1. SCOTCH preprocessing pipeline is capable to handle data generated by both 10X Genomics and Parse Biosciences libraries. For the 10X Genomics libraries, SCOTCH detects polyA/T tails connected to UMI and cell barcodes, and account for possible read truncations at the 5’ end. For the Parse libraries, SCOTCH similarly accounts for possible 5’ read truncations if polyA/T tails can be detected in the long reads; otherwise, it considers read truncations possibly happen at both 3’ and 5’ ends when hexamer primers might be used (Figure 1a). SCOTCH takes BAM files with tagged barcodes generated by vendor-supplied pipelines (wf-single-cell and parse) as input to align reads to known and novel isoforms, which are treated as combinations of known exons (Figure 1b–c, Methods). Specifically, SCOTCH encodes reads by the presence or absence of each exon, using read-exon mapping percentages. It rejects read-isoform mappings if (1) the isoform annotation includes any exons that the read skips or (2) the isoform annotation fails to encompass all exons covered by the read. For reads unmappable to any known isoforms, SCOTCH constructs novel read graphs and identifies novel isoforms at the pseudo-bulk level using the Louvain clustering method. These reads are then aligned to novel isoform annotations. Note that, we leverage the unique mapping reads at the pseudo-bulk level to handle reads mapping to multiple isoforms.

After preprocessing lr-scRNA-seq data, we aim to perform analysis with transcript-level resolution to unveil alterations obscured by conventional differential gene expression analysis. Differential transcript usage (DTU), which is defined as variations in relative abundance of transcripts of the same gene across different conditions or cell types, explain changes in phenotype between cell types, tissues, or disease cohorts^24–26^. The SCOTCH statistical pipeline, which is inspired by Longcell^17^ for exon-inclusion analysis and LIQA^27^ for truncation handling, can be used to identify DTU on both the gene and transcript levels (Figure 1d, Methods). For each gene, SCOTCH estimates the average transcript usage of a cell population by fitting a Dirichlet-multinomial distribution, which captures inter-cell variations quantified by the over-dispersion parameter *ϕ* This parameter is mean invariant, where small values indicate cells with similar isoform co-expression patterns, and large values suggest a more exclusive expression mode, with each cell predominantly expressing one isoform while different cells may express others. Employing likelihood ratio tests, we assess whether transcript usage differ between two cell populations at the gene level, and we examine whether a specific transcript is differentially utilized at the transcript level. One special case of transcript usage alteration is isoform switching, here defined by changes of the dominant isoform between two cell populations (it may also be defined as a synonym for DTU in some publications). SCOTCH is designed to pinpoint isoform switching events, whose effect size is measured by the absolute sum of differences in the dominant isoform proportions between the two cell populations.

To benchmark our methods and assess how lr-scRNA-seq can be used to infer novel biological insights that are unattainable through traditional short-read sequencing approaches, we generated a substantial amount of evaluation data for two human PBMC samples using both 10X Genomics and Parse libraries, Illumina and nanopore sequencings (Table 1). Throughout the study, we first demonstrated that the availability of R10 flowcells facilitates isoform-level analysis without paired short reads or complex computational methods previously required to handle high sequencing error rates. This is shown through several key comparisons, including short-read versus long-read sequencing, R9 versus R10 flowcells sequencing technologies, and the 10X Genomics versus the Parse Bioscience library preparation system. Further into the study, we analyzed lr-scRNA-seq data on human peripheral blood mononuclear cells (PBMC) to showcase the effectiveness of SCOTCH’s statistical framework in analyzing differential transcript usage, detecting isoform switching events, and assessing inter-cell heterogeneity.

### R10 flowcells facilitate isoform-level analysis with improved sequencing quality

When preprocessing lr-scRNA-seq data with library constructed on the 10X Genomics platform, a threshold of edit distance (ED) is set for UMI clustering and cell barcode resolution. This ED threshold determines the stringency of the error correction steps for UMIs and cell barcodes, directly impacting the precision of read allocation to particular cells and the effectiveness of molecule de-multiplexing. The conventional wisdom is that R9 flowcells need an ED of 2 for accurate read-cell mapping^28^, owing to their higher sequencing errors compared to short-read sequencing. However, the newer R10 flowcells display improved sequencing quality, particularly in homopolymers regions^29^, where they have lower deletion rates in thymine and adenine sequences than R9, albeit with increased mismatches^30^. The R10’s ability to match Illumina’s sequencing quality standards yields flexibility in choosing between ED1 and ED2, allowing for more efficient and accurate read mappings to the corresponding cells.

**Figure 2.**
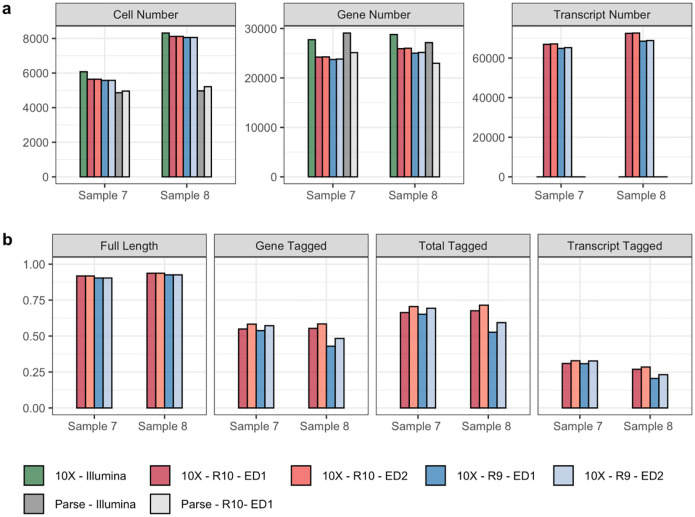
Influence of edit distance (ED) criteria on final results of five single-cell library + sequencing platforms generated by vendor computational pipeline: 10X + Illumina, Parse + Illumina, 10X + Nanopore_R9, 10X + Nanopore_R10, Parse + Nanopore R10. (**a**) The number of cells, genes and transcripts that are identified based on different flowcell versions and different ED thresholds (1 or 2). (**b**) The fraction of reads that are classified as full-length, gene-tagged, total-tagged, and transcript-tagged in two samples sequenced by both R9 and R10 flowcells on the Nanopore platform.

To empirically substantiate the technical advancements offered by R10 flowcells that facilitate isoform-level analysis, we systematically examined and compared sequencing statistics across different combinations of single-cell libraries and sequencing technologies (10X + Illumina, Parse + Illumina, 10X + Nanopore_R9, 10X + Nanopore_R10, Parse + Nanopore_R10) that are preprocessed by vendor-supplied computational pipelines (Table S1, Figure 2). Regardless of whether an ED of 1 or 2 is applied, R10 flowcells align more closely with short-read sequencing in terms of median numbers of genes and UMIs per cell (Table S1), as well as the total counts of cells, genes, and transcripts (Figure 2a). R10 flowcells also show higher proportions of reads categorized as full length, total-tagged, gene-tagged, and transcript-tagged (Figure 2b), indicating higher sequencing quality. When applying an ED of 2, both R9 and R10 flowcells display an increase in sequencing saturation and elevated median gene, transcript, and UMI counts per cell compared to using ED of 1. This is because a higher ED threshold leads to more lenient clustering, thereby enabling the recognition of more unique transcripts. Notably, the increase seen with ED2 relative to ED1 is less pronounced for R10 than R9, and both ED1 and ED2 exhibit comparable performance in terms of the total numbers of cells, genes, and transcripts for R10 (Figure 2a), suggesting that the improved sequencing accuracy of R10 flowcells reduces the need for a higher ED for reliable transcript identification. Additionally, R10 consistently outperforms R9 in sequencing saturation, and median numbers of genes, transcripts, and UMI counts per cell across both ED settings, affirming its ability to capture a more comprehensive transcriptome profile even at a stricter ED threshold.

### SCOTCH facilitates single-cell transcript usage analysis

**Figure 3.**
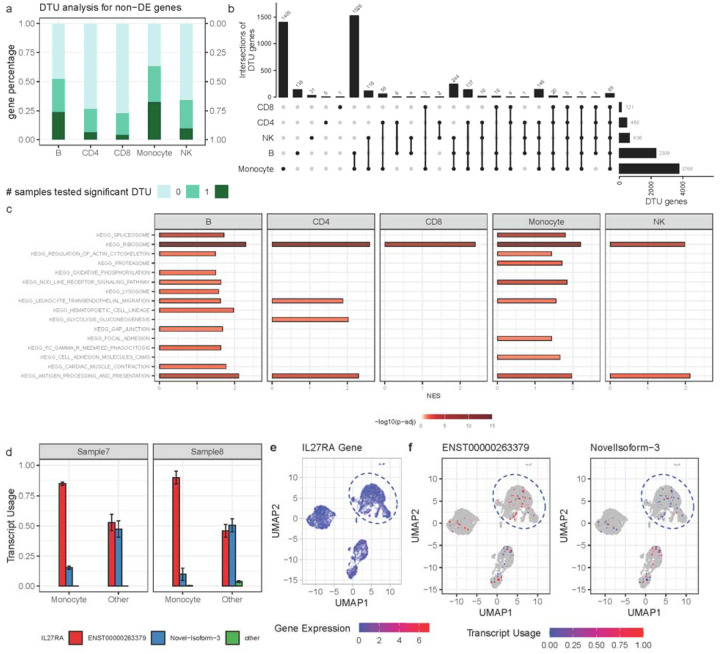
Single-cell transcript usage analysis using the SCOTCH pipeline. (**a**) Proportions of non-differentially expressed genes with differential transcript usage (DTU) in various cell types as analyzed by the SCOTCH pipeline. The darkness of colors indicates the number of samples in which significant DTU was observed. (**b**) Upset plot displaying the intersections of significant DTU genes of each cell type in against others in both sample 7 and sample 8. (**c**) Pathway enrichment analysis of significant DTU genes for each cell type. x-axis indicates normalized enrichment score (NES). The darkness of colors indicates the significance level of enrichment analysis. (**d**) Bar plot displaying the proportions of transcript usage for *IL27RA* isoforms of ENST00000263379, Novel-Isoform-3, and other isoforms comparing monocytes and other cell types for sample 7 and sample 8. (**e**) UMAP visualization for expression levels of the *IL27RA* gene. (**f**) UMAP visualization for *IL27RA* isoform ENST00000263379 and Novel-Isoform-3 usages. Cells within blue circles are monocytes.

The results above suggest that with technical improvements of R10 flowcells, computational methods should shift focus from managing high sequencing error rates to in-depth transcriptome analysis with isoform-level characterization. Investigating transcript usage across diverse cell populations can provide unique biological insights beyond mere gene expression comparisons. In this context, we employed SCOTCH preprocessing and statistical pipeline to analyze lr-scRNA-seq data from two human PBMC samples sequenced with 10X + Nanopore_R10 protocol applying an ED of 1. We classified cell types and performed conventional differential gene expression (DGE) analysis by comparing each cell type against the others using the gene-level count matrix. We then identified genes with DTU using the transcript-level count matrix. Figure3a reveals that between 4 to 32 percent of the genes, despite not being differentially expressed between cell populations, exhibit DTU in both samples, while 13 to 63 percent of these genes display DTU in at least one sample. These findings suggest that relying solely on gene expression comparisons could overlook significant biological variations introduced by DTU. Figure 3b illustrates the number of DTU genes identified in both sample 7 and sample 8, highlighting differential transcript usage as a common feature among various cell types. Monocytes exhibit the highest number of DTU genes at 3766, with 1405 unique to this cell type, suggesting that distinct isoforms may play roles tied to monocyte-specific functions. B cells have the second highest DTU gene number at 2309, with 1526 overlapping with monocytes, signaling shared transcriptomic characteristics and pathways (Figure 3c). NK cells, along with CD4, and CD8 T cells exhibit a lower DTU gene count, which could be related to their lower prevalence in the samples. These findings underscore the cell-type-specific nature of transcript usage. We then conducted gene set enrichment analysis to explore the biological processes relating to cell type-specific transcript usage patterns (Figure 3c). We observed that certain biological processes are consistently enriched in most cell types, such as ribosome, leukocyte trans-endothelial migration, and antigen processing and presentation. Monocytes and B cells show the most similar pathway enrichment patterns compared to other cell types, echoing the substantial overlap of DTU genes between them. Such parallels reinforce the shared transcriptomic features and functional pathways, highlighting the intricate connection between transcript usage and cellular function in the immune system.

### SCOTCH uncovers transcriptomic variations and catalogs novel isoforms

To showcase the utility of SCOTCH in identifying DTU genes, especially when gene expression levels are unchanged, we examined the IL27RA gene, which encodes a component of the receptor for interleukin-27 (IL-27) and plays critical roles in immune cell differentiation and the control of cytokine production^31^. The exploration of *IL27RA* isoforms has therapeutic relevance, as a soluble form of IL-27Rα has been shown to act as a natural antagonist to IL-27^32^. Manipulating the expression of various isoforms could provide novel methods to modulate immune responses in diseases influenced by IL-27. As shown in Figure 3d, transcript usages are markedly different for the IL27RA gene (p-adj < 0.0001) and two of its isoforms (ENST00000263379: p-adj < 0.0001, Novel-Isoform-3: p-adj < 0.0001) between monocytes and other cell types in both sample 7 and sample 8. In particular, the isoform ENST00000263379 is predominant in monocytes with estimated transcript usage being 0.85 for sample 7 and 0.90 for sample 8 (Figure 3d, Figure 3f), respectively. Conversely, in other cell types, the usage of ENST00000263379 is nearly equivalent to that of Novel-Isoform-3 (Figure S1), with the latter accounting for half the isoform population in sample 8, indicative of isoform switching (effect size 0.85). Despite these differences, the overall expression levels of the *IL27RA* gene remain unchanged between monocytes and other cell types, with a p-adj value of 1 (Figure 3e). These results suggest that SCOTCH is adept at uncovering transcriptomic variations and cataloging novel isoforms, even when there are no changes in overall gene expression levels.

**Figure 4.**
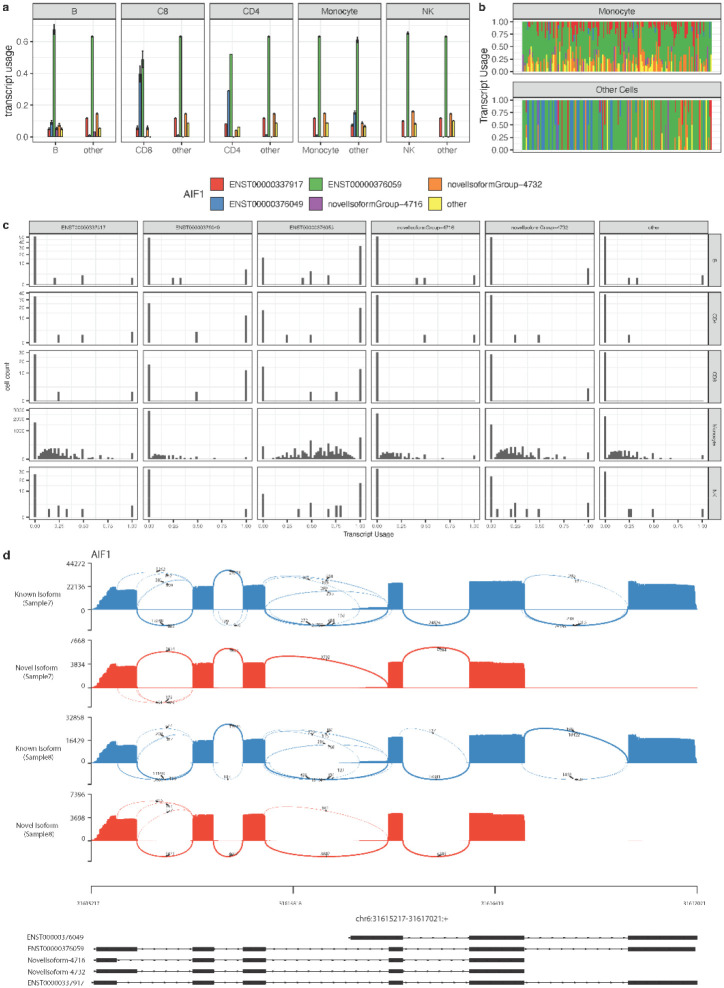
A typical example of using the SCOTCH pipeline to analyze differential transcript usage (DTU) for the *AIF1* gene. (**a**) Bar-plot of cell-type specific average transcript usage inferred by SCOTCH. (**b**) Distribution of transcript usage of monocytes and all other cell types. Each column represents a cell, with colors indicating isoform compositions used within each cell. Monocytes are downsampled to the same number of other cells. (**c**) Histograms of transcript usage distribution for five cell types with diverse co-expression patterns. (**d**) SCOTCH pipeline identifies novel isoforms for the *AIF1* gene across different samples. Displayed is sashimi plot for reads mapped to known *AIF1* isoforms (ENST0000376049, ENST00000376059, ENST00000337917) and novel isoforms (novelisoform-4716, novelisoform-4732), where two novel isoforms do not contain the last exon on the 3’ end. All results shown are based on 10X-ONT PBMC data of sample 8.

We further examined the *AIF1* gene as another case study to elucidate the utility of SCOTCH in DTU analysis. *AIF1* is instrumental in cytoskeletal rearrangements and cell migration^33^. In immune responses, *AIF1* acts as a critical modulator of inflammation and immune cell activation^34^. *AIF1* isoforms may vary in their expression patterns, subcellular localization, or interaction partners, influencing the roles of *AIF1* in various cellular processes, including inflammation and immune responses^35^. Figure 4a demonstrates DTU patterns for the *AIF1* gene of sample 8 when comparing each cell type against others. Among the five isoforms that are commonly detected, two are novel, with ENST00000376059 being the predominant isoform in all five cell types, succeeded by ENST00000376049 or Novel-Isoform-4372 in different cell types. On the gene level, significant DTU was observed in all cell types except NK cells (Monocytes: p-adj < 0.0001; B cells: p-adj = 0.0005; CD4 T cells: p-adj < 0.0001; CD8 T cells: p-adj < 0.0001; NK cells: p-adj = 0.963). On the transcript level, isoform ENST00000376049 was notably prevalent in CD4 and CD8 T cells (p-adj < 0.0001 for each), with the corresponding average transcript usage (TU) being 0.29 and 0.40, versus 0.01 for other cells. On the other hand, ENST00000376049 was found to be rare in monocytes (TU = 0.01) but relatively prevalent in others (TU = 0.15, p-adj < 0.0001). In addition to assessing differences in transcript usage across cell populations, SCOTCH also elucidates isoform co-expression patterns using the mean-invariant over-dispersion parameter *ϕ* To account for the imbalance in the numbers of cells expression *AIF1* isoforms, we down-sampled monocyte data to match the number of cells from other types, enabling a visually comparable analysis of cell type-specific isoform co-expression. Figure 4b shows that monocytes commonly express different isoforms simultaneously with similar usage proportions (*ϕ* = 0.01), whereas other cells exhibit more heterogeneity, often exclusively expressing a single isoform, as indicated by a higher dispersion value of *ϕ* = 0.08. Figure 4c further shows the distribution of transcript usage within individual cells of a cell type, revealing the variabilities of isoform dominance and co-expression patterns across different cell types. These diverse isoform usage patterns have implications for the intricate roles of *AIF1* in cellular and immune processes.

One unique advantage of SCOTCH pipeline over conventional wf-single-cell pipeline provided by the vendor is its capability of identifying novel isoforms. About 35% of reads align with known transcripts, while 20% are categorized as novel isoforms (Table S2), among which about 70% are consistently detected across both samples 7 and 8. Figure 4d exemplifies this advantage, showcasing the detection of Novel-Isoforms-4716 and Novel-Isoforms-4732 of the *AIF1* gene in both samples 7 and 8. These novel isoforms are characterized by distinct splicing patterns that differ from those of the known isoforms. Notably, these novel isoforms do not have the last exon included in the known isoforms of ENST00000376049, ENST00000376059, and ENST00000337917. Instead, they directly connect to the polyadenylation site (polyA tail), resulting in transcripts with alternative terminal sequences. The SCOTCH pipeline’s ability in uncovering novel isoform structures underscores its potential to yield deeper insights into the complexity of the transcriptome.

### Consistency and reproducibility between different sequencing technologies, single-cell libraries, and computational pipelines

**Figure 5.**
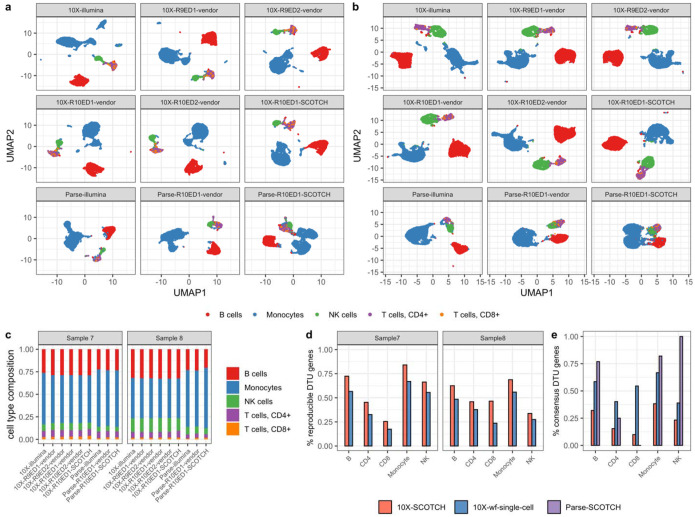
Consistency and reproducibility analysis comparing various technical platforms and pipelines. (**a-b**) UMAP visualization for clustering of different cell types comparing between short-read and long-read scRNA-seq, 10X Genomics and parse single-cell libraries, R9 and R10 flowcells, Edit Distance of 1 and 2, as well as vendor and SCOTCH pipelines on the same PBMC samples. (**a**) PBMC sample 7. (**b**) PBMC sample 8. (**c**) Cell type compositions for sample 7 in a and sample 8 in b. (**d**) Percentage of DTU genes that are also identified in the other sample analyzed by SCOTCH and wf-single-cell pipelines. (**e**) Percentage of DTU genes identified by 10X-SCOTCH, 10X-wf-single-cell, and parse-SCOTCH that are also identified by the other pipeline of 10X-wf-single-cell, 10X-SCOTCH, and 10X-SCOTCH, respectively.

We have demonstrated technical enhancements of lr-scRNA-seq with R10 flowcells and we leveraged the SCOTCH pipeline for transcript usage analysis above. We next sought to determine the consistency and reproducibility of the results obtained from the SCOTCH pipeline with data generated by various libray construction and sequencing protocols. To this end, we investigated the concordance of analytical outcomes across an array of technical platforms and pipelines. We first compared cell type clusters on the gene level across short-read and long-read scRNA-seq, 10X Genomics and parse single-cell libraries, R9 and R10 flowcells, and with Edit Distance of 1 and 2, as well as between vendor-specific pipelines and SCOTCH, utilizing PBMC samples (Figure 5a–c). The cell type clusters were generally consistent in UMAP visualizations and cell type proportions across different platforms and analytical methods, with monocytes being the most prevalent cell type, followed by B cells. These patterns were reproducible across two samples, underscoring the reliability of cell type identification across diverse sequencing technologies, library preparations, and analytical approaches on the gene level. On the transcript level, we assessed the number of DTU genes across two samples to compare the wf-single-cell and SCOTCH pipelines, utilizing data generated by 10X Genomics library with nanopore R10 flowcells with an ED setting of 1. As shown in Table 2, the 10X-SCOTCH consistently identified a greater number of DTU genes across different cell types than the 10X-wf-single-cell pipeline, with increased reproducibility demonstrated by a second sample, as depicted in Figure 5d. Over 50% of DTU genes identified by 10X-wf-single-cell pipeline were validated by 10X-SCOTCH in both samples (Figure 5e). Note that, given that monocytes are more abundant, it is anticipated that a higher number of cell type-specific DTU genes would be detected for monocytes than for other cell types. Owing to the low sequencing depth of the Parse library, we proceeded to determine whether the Parse platform enables the identification of key DTU genes that were also detected using the 10X Genomics platform. As displayed by Figure 5e, over 75% of DTU genes detected in B, monocytes, and all those of NK cells by Parse-SCOTCH were validated by 10X-SCOTCH, suggesting strong consensus between both single-cell library construction methods.

### SCOTCH statistical pipeline aids in revealing biological insights beyond gene expression

Substantial isoform variations revealed by our study offer new perspectives on their roles in immune regulation and response. In our analysis, we have unveiled a significant number of genes with DTU across various cell types (Table 2), with a pronounced finding in monocytes. We observed significant DTU across 1,441 genes, corroborated in two samples through dual analytical pipelines of 10X-wf-single-cell and 10X-SCOTCH. Among these genes, a number of genes related to neutrophil mediated immunity, including *CD44, CD63, CSNK2B, DEGS1, HMGB1, ITGB2, PTPRC, RAB27A, RAP1B*, and *RHOA*, have been particularly highlighted in the literature for their functional significance by differential isoforms^36–40^. Monocytes play a multifaceted role in this immunity aspect, capable of differentiating into macrophages or dendritic cells in response to pathogens or inflammatory signals, thereby releasing cytokines and chemokines that stimulate granulocyte activation^41^. Additionally, monocytes can directly interact with granulocytes through cell-to-cell contact and signaling pathways, thereby modulating their activation and recruitment to sites of infection or inflammation. These genes offer extensive insights into the intricate mechanism monocytes employ in neutrophil-mediated immunity. The genes and DTUs identified in our study suggest that the interplay between monocytes and granulocytes involves a complex network of molecular interactions mediated by various genes. For example, specific isoforms of *CD44, ITGB2*, and *RAP1B* facilitate the adhesion and migration of monocytes to inflammatory sites, where they release pro-inflammatory cytokines such as *IL-18* and *HMGB1*, and contribute to granulocyte activation^42, 43^. Additionally, isoforms of *CD63* and *RAB27A* regulate granule protein sorting, vesicle trafficking, and secretion of inflammatory mediators^44^, further modulating monocyte-granulocyte interactions.

In B cells, significant DTU was identified in 741 genes by both 10X-wf-single-cell and 10X-SCOTCH, notably those involved in antigen processing and presentation, such as the *HLA* genes and *CD74*, highlighting the pivotal role of variations of transcript usage in modulating adaptive immune responses. Transcript usage variation of genes involved in antigen processing and presentation by B cells plays a pivotal role in modulating adaptive immune responses. For instance, the *HLA-DMB, HLA-DPA1, HLA-DPB1, HLA-DQA1*, and *HLA-DQB1* genes, encode *MHC* class II alpha and beta chains, which determine peptide-binding specificities and antigen presentation capabilities. Transcript usage variation in these genes could alter the repertoire of presented antigens and influence immune responses^45^*. HLA-E* and *HLA-F* encode non-classical MHC class I molecules, and participate in immune regulation and antigen presentation^46^. Isoform variation may modulate their interactions with immune receptors on B cells, impacting antigen presentation pathways. *CD74*, a key player in MHC class II trafficking and peptide loading, undergoes alternative splicing, potentially influencing its role in antigen presentation efficiency^47^. The widespread identification of these genes with DTU, especially those common in monocytes and B cells, underscores the significant influence of isoform diversity on the functional complexities of immune cells.

## Discussion

In this study, we first generated benchmarking datasets of two human PBMC samples and demonstrated the technical advancements of R10 flowcells through comparing of five different technical sequencing approaches: 10X + Illumina, 10X + ONT_R9, 10X + ONT_R10, Parse + Illumina, and Parse + ONT_R10, under ED settings of 1 and 2 (Table 1). These advancements have allowed us to shift the focus of computational tools to unraveling the complexities of the transcriptome using lr-scRNA-seq. As a result, we introduced SCOTCH, a suite of computational and statistical pipelines designed for the processing and analysis of lr- scRNA-seq data. The preprocessing pipeline of SCOTCH is compatible with single-cell libraries from both the 10X Genomics and Parse Biosciences platforms. Notably, the Parse Biosciences platform has a unique advantage as it allows the simultaneous sequencing of multiple samples, obviating the need for further data integration and facilitating the analysis of special cell populations. Moreover, the preprocessing pipeline handles multiple-mapping issues in reads and is adept at robustly identifying novel isoforms at the pseudo-bulk level. The statistical pipeline of SCOTCH facilitates the comparison of transcript usage between cell populations, the identification of isoform switching events, while also providing insights into isoform co-expression patterns. By leveraging full-length transcript information from lr-scRNA-Seq data, SCOTCH will significantly improve our understanding of cell type-specific transcriptomic alterations, encompassing both gene expression alterations and isoform usage preferences. This enhancement will deepen our insights into cell type specificity across various biological processes and disease physiologies.

The introduction of R10 flowcells has been a significant advancement, and vendor-supplied tools like the wf-single-cell pipeline for 10X Genomics and the Parse pipeline for Parse Biosciences are adequate for barcode identifications. Nonetheless, for subsequent preprocessing step, SCOTCH exhibits several notable technical improvements and design considerations compared to vendor-supplied computational methods that we wish to discuss here. Firstly, the Parse pipeline, the only available tool for Parse Biosciences data currently, is designed purely for conventional gene-level expression analysis and lacks the functionality to assign reads to corresponding gene isoforms. In contrast, SCOTCH leverages the full-length transcripts information from lr-scRNA-seq to precisely align reads to gene isoforms, which is crucial for downstream isoform usage analysis. Secondly, several studies have demonstrated that long-read sequencing can identify novel isoforms from bulk and single-cell samples^48, 49^. While the wf-single-cell pipeline does offer alignment to the transcript resolution, it relies on known transcript annotations to quantify transcript counts without the capability to identify novel isoforms. SCOTCH, on the other hand, detects and annotates candidate novel isoforms from reads that cannot be confidently assigned to any existing isoforms on the pseudo-bulk level. It then performs read-isoform alignments to ensure the robustness of the identified novel isoforms. In addition, SCOTCH introduces a statistical framework tailored for transcriptome analysis. In our method to identifying differential isoform usage, rare isoforms are aggregated under a category termed “other”, thereby limiting the number of isoforms used in statistical tests. This approach effectively balances the discovery of novel isoforms with the detection of biologically significant isoforms.

We also acknowledge several limitations inherent to the SCOTCH pipeline and our study. A primary issue involves multi-mapping reads, where a single read may align to multiple isoforms of a gene. SCOTCH’s current strategy estimates mapping probabilities using uniquely mapped reads and a multinomial distribution to assign these reads to isoforms. Although this is computationally efficient, it can potentially introduce bias if the uniquely mapped reads aren’t fully representative of the overall mapping probabilities. An improvement on this process could take inspiration from the LIQA method^27^, which weights reads based on length and alignment quality, thus compensating for possible read truncation and enhancing read-isoform mapping precision. Secondly, SCOTCH’s capability to identify novel isoforms is dependent on existing exon annotations and, consequently, it is not designed to recognize isoforms that feature novel exonic structures like intron retentions or alternative splice sites absent in existing databases. This gap suggests an opportunity for future advancements in SCOTCH’s design to enable the discovery and annotation of completely new exon configurations. Moreover, SCOTCH’s statistical framework is primarily geared towards detecting shifts in transcript usage at the gene or isoform levels. An area ripe for expansion is the incorporation of analyses for local splicing variations^11^, which would make SCOTCH a more comprehensive tool for transcriptome analysis. Lastly, a set of five samples was pooled by the Parse single-cell library construction approach and subsequently sequenced using R10 flowcells, yet only two of them are used in the current study. Given the relatively low sequencing depth per sample, direct comparisons between the Parse results and those from the 10X Genomics library cannot be completed in the current study. However, we have demonstrated consistency in the detection of differentially used transcripts (DTUs) when there is an adequate number of cells and sufficient coverage.

In the evolving field of single-cell and single-nucleus sequencing, various limitations are present within current protocols and computational methodologies, yet they also offer significant opportunities for advancement. A crucial limitation is the challenge of analyzing full-length mRNA isoforms. This analysis is vital for the detailed profiling of cellular subtypes and the detection of transcript variants such as alternative splicing or alternative polyadenylation sites^5^. With emerging tools like SCOTCH and improvements in vendor-supplied computational pipelines, the depth of our insights into complex gene expression patterns is set to increase. Additionally, high dropout rates significantly impede the accuracy of gene expression profiling^50^, an issue that is particularly pronounced in long-read transcriptome analysis at the transcript level. A promising direction for future pursuing could involve the development of computational methods specifically tailored for long-read data to mitigate this issue, building on approaches that have shown effectiveness in short-read data^51–53^. Furthermore, non-polyA RNA, which includes various functionally important regulatory RNAs^54^, is better captured by the Parse Biosciences protocol due to its use of a mix of polyA primers and random hexamers, unlike the 10X Genomics protocol that requires a polyA tail exclusively. Future experimental approaches could further refine these techniques to provide a more comprehensive analysis of the transcriptome, ensuring that even non-polyadenylated RNA molecules are accurately represented. Additionally, future improvements are poised to refine unsupervised clustering utilizing full-length transcript information, leading to enhanced resolution at the transcript level and deeper biological insights. Another exciting frontier is the assessment of RNA velocity through long-read sequencing data^55^, which holds the potential to drastically alter our understanding of RNA dynamics and regulatory mechanisms. Lastly, advancements in sequencing technology that increase depth and reduce costs are expected to facilitate more precise allele-and isoform-resolution counting, thereby broadening the applicability of transcriptomic analyses across diverse cell populations. This progression promises a more detailed and nuanced understanding of transcriptomics in health and disease. Collectively, these efforts are critical for refining lr-scRNA-seq analyses and advancing comprehensive cellular profiling.

In summary, by capturing the repertoire of transcriptional isoforms across diverse cell types, SCOTCH provides a foundation for delving into the functional significance of these isoforms in various cell types, and holds promise in unraveling the complexity of cellular processes.

## Methods

### Preparation of the PBMC samples

Blood samples from two de-identified individuals, one male and one female, were collected using tubes coated with EDTA. These samples were promptly processed to separate PBMCs through Ficoll density gradient centrifugation at the Center for Applied Genomics (CAG) at the Children’s Hospital of Philadelphia (CHOP). The Institutional Review Board at the CHOP approved this study.

### Single cell library preparation on the 10X Genomics platform

RNA samples were processed using 10X Genomics Next GEM Single Cell 3’ Kit (V3.1) following manufacturer recommended protocols. During 10x library preparation, an aliquot of about 10ng of full-length amplified barcoded cDNA was taken for Nanopore sequencing. The remaining amount was processed further using the standard 10X protocol for Illumina sequencing.

### Single-cell library preparation on the Parse Biosciences platform

RNA samples were processed using Parse Bioscience Evercode WT Mini v2 Kit following the manufacturer recommended protocol. During the first barcoding step, two control samples were distributed across 3 wells, Sample 7 and Sample 8 were distributed across 4 wells each, and a PBMC control was added to the last well. Approximately 70ng of each sublibrary was taken for Nanopore sequencing while remaining amount was used for Illumina sequencing.

### Oxford Nanopore sequencing of single-cell libraries

The single cell cDNA was processed for Nanopore sequencing using the Oxford Nanopore Technology (ONT) Ligation Sequencing Kit V14 (ONT, SQK-LSK114), cDNA-PCR Sequencing Kit (ONT, SQK-PCS111), or a combination of the cDNA-PCR Sequencing Kit and the Rapid Sequencing Kit (ONT, SQK-RAD114), and two custom primers, 5′-/5Biosg/CAGCACTTGCCTGTCGCTCTATCTTCCTACACGACGCTCTTCCGATCT-3′ and 5′-CAGCTTTCTGTTGGTGCTGATATTGCAAGCAGTGGTATCAA CGCAGAG-3′. The primers target the Illumina Read 1 sequence and the 10X Genomics template switch oligo sequence. 10ng of the single cell cDNA was mixed with 10 uM of both custom primers and LongAmp Hot Start Taq 2X Master Mix (NEB, M0533S). Amplification in a thermal cycler was performed using the following condition: 94C for 3 minutes, four cycles of 94C for 30 seconds, 66C down to 58C for 40 seconds, 58C for 50 seconds, and 65C for 6 minutes, a final extension cycle of 65C for 10 minutes, and a hold of 4C. Clean up was performed using AMPure XP beads (Beckman Coulter^™^ cat # A63881) with a 1.25x solution to beads ratio, and washed twice with freshly made 200 ul of 80% ethanol, and the amplified cDNA was eluted with 10 ul of nuclease-free water.

Full-length cDNA was isolated using M280 streptavidin, 10 ug/ul (Invitrogen, 11205D), which the biotinylated cDNA binds to. 4 ml of a 2X wash/bind buffer was prepared with 10 mM Tris-HCl pH 7.5, 2 M NaCl, and 1 mM EDTA. Half of the 2X wash/bind buffer was used to make a 1X wash/bind buffer to us as a wash buffer. A 5 ug/ul streptavidin bead was made by replacing the buffer from 5 ul of the streptavidin beads with 10 ul of the 2X wash/bind buffer. 10 ul of the biotinylated cDNA was added to the 5 ug/ul beads and incubated at room temperature for 20 minutes. The mixture was then washed thrice with 1 ml of the 1X wash/bind buffer. A final wash was performed using 200 ul of 10 mM Tris-HCl pH 7.5. The beads were then resuspended in 20 ul of nuclease-free water.

The isolated full-length cDNA was then amplified with PCR. The 20 ul of the amplicon-bead conjugate was added to a 30 ul mixture of 10 uM PCR primer, LongAmp Hot Start Taq 2X Master Mix (NEB, M0533S), and nuclease-free water. The PCR primer used was dependent on the flow cell and/or sample used. For the R9 we used cPRM from SQK-PCS111, for the R10 for Sample7 and Sample8 we used cPRM from SQK-PCS111. Amplification in a thermal cycler was performed using the following condition: 94C for 3 minutes, four cycles of 94C for 15 seconds, 56C for 15 seconds, and 65C for 6 minutes, a final extension of 65C for 10 minutes, and a hold of 4C. Clean up was performed using AMPure XP beads (Beckman Coulter^™^ cat # A63881) with a 1.25x solution to beads ratio, and washed twice with freshly made 200 ul of 80% ethanol, and the amplified cDNA was eluted with 15 ul of nuclease-free water.

For the samples processed with a PRM primer, an additional end-prep step needs to be performed before the adapter can be ligated to the full-length transcripts. 200 fmols from the previous step was mixed with the Ultra II End-prep Reaction Buffer and Ultra II End-prep Enzyme Mix (NEB, E7546) and incubated at 20C for 5 minutes and 65C for 5 minutes. Clean up was performed as previously, but with a 1.0X sample-to-beads ratio and eluted with 60 ul of nuclease-free water.

For the R9 and Sample7 and Sample8 R10 samples Rapid Adapter T was added to 35 fmol of cDNA and incubated at room temperature for 5 minutes. For the remaining R10 samples, the end-prepped cDNA was added to a mixture of Ligation Buffer (LNB), NEBNext Quick T4 DNA Ligase, and Ligation Adapter (LA). The mixture was then incubated for 10 minutes at room temperature. Clean up was done with a 2.5x sample-to-beads ratio, washed twice with 250 ul of the Short Fragment Buffer (SFB), and eluded with 25 ul of Elution Buffer.

The Parse samples were processed for Nanopore sequencing using the Ligation Sequencing Kit V14. The libraries were end repaired by mixing the Parse samples with the Ultra II End-prep Reaction Buffer and Ultra II End-prep Enzyme Mix and incubated at 20C for 5 minutes and 65C for 5 minutes. Clean up was performed with a 1.0X sample-to-beads ratio and eluted with 60 ul of nuclease-free water. To add the Nanopore adapter to the cDNA, the end-prepped cDNA was added to a mixture of Ligation Buffer (LNB), NEBNext Quick T4 DNA Ligase, and Ligation Adapter (LA). The mixture was then incubated for 10 minutes at room temperature. Clean up was done with a 2.5x sample-to-beads ratio, washed twice with 250 ul of the Short Fragment Buffer (SFB), and eluded with 25 ul of Elution Buffer.

Samples are loaded into an R9 or R10 PromethION flowcell (FLO-PRO002 or FLO-PRO114M) using the standard Nanopore loading protocol that involves priming the flow cell twice with a flow cell flush and flow cell tether mixture and then adding the Nanopore library that has been mixed the sequencing buffer and loading beads. Libraries were sequenced on a P2-solo and 96 hours.

### Nanopore data processing

Sequencing data was basecalled using Guppy v6.5.7 with super-accuracy (SUP) model. After basecalling, cell identification based on UMI barcodes and gene and transcript count matrices generation was done using Nanopore’s wf-single-cell v1.0.1 (https://github.com/epi2me-labs/wf-single-cell) pipeline. To show improved accuracy between the R9 and R10 flowcells, the pipeline was run twice with the R9 and R10 data for sample 7 and sample 8. Once with a barcode edit distance of 1 and a second time with a barcode edit distance of 2. Barcode edit distance is the allowance of a specified number of errors a barcode can have to be classified as a valid cell barcode. To use the Nanopore data on the Parse pipeline, the Nanopore reads would have to be split into artificial paired-end reads. With read 2 containing the Parse barcode sequences and read 1 containing the rest of the reads. Minimap2 was used for alignment instead of STAR.

### Cell clustering, visualization and cell type assignment

The processed sequencing data were analyzed using the Seurat R package^56, 57^, which facilitated the processing, normalization, and identification of cell clusters based on gene expression patterns. Cell subtyping was performed utilizing singleR and the celldex::DatabaseImmuneCellExpressionData() function^58^. For visual representation and dimensionality reduction, Uniform Manifold Approximation and Projection (UMAP)^59^ was applied. The Python package of Trackplot^60^ was used for generating sashimi plots.

### The SCOTCH pipeline for isoform level characterization of single cell RNA-seq data

The SCOTCH pipeline requires two types of input files: a long-read RNA-seq file provided as an annotated BAM file, and an isoform annotation file in GTF format. Upon processing these inputs, SCOTCH generates count matrices at both the gene level and the transcript level, with the capability to identify novel isoforms do not present in the original annotation file. Leveraging these gene and transcript count matrices, SCOTCH enables statistical analyses of the transcriptome across different cell populations, including the detection of genes with differential isoform usage and the identification of isoform switching events. SCOTCH is available at http://github.com/WGLab/SCOTCH. Detailed description of the computational methods are given below.

1. Generate annotated bam files: Depending on the library type, we utilize either the ‘wf-single-cell’ (10X Genomics libraries) or ‘parse’ (Parse libraries) pipeline for read alignment and barcode processing. This process produces BAM files annotated with barcode information.
2. Generate gene annotation files: Utilizing GTF gene annotation files, we segment exons of each gene into distinct, non-overlapping sub-exons. We compile detailed gene information, including gene name, strand direction, genome locations, sub-exon coordinates, and transcript annotations as combinations of sub-exons. This compiled information is stored in the pickle file format. Additionally, to enhance the efficiency of mapping reads to genes, we group genes sharing overlapping regions into non-overlapping meta-genes.
3. Assign reads to genes: For each meta-gene, which may consist of either a single gene or multiple overlapping genes, we examine reads that fall entirely the genomic region. In the case of duplicated reads resulting from PCR amplifications, as identified by UMI or sub-library barcode information, we retain only the longest read for further analysis. Given that a read can map to multiple genes due to their overlapping nature, our strategy prioritizes mapping the read to the gene where it aligns with an exon. If the read still maps to multiple genes, we adhere to the following hierarchy: first, to genes where the read matches an existing isoform; second, to genes corresponding to a novel isoform; and lastly, to genes where the read aligns with exons but cannot be categorized as known or novel isoforms.
4. Assign reads to annotated isoforms: Isoforms are treated as specific combinations of non-overlapping sub-exons. We first align the read to sub-exons exceeding 20bp in length. A read is considered aligned to the sub-exon if it covers more than 80% of the sub-exon’s length, a read is deemed to have skipped the sub-exon if it fails to cover more than 20% of its length, otherwise as ambiguously alignment. The read is initialized as mapped to all annotated isoforms, and we exclude any read-isoform pairings if: (1) the read encompasses sub-exons not contained in the annotated isoform, or (2) the read skips sub-exons that are present in the annotated isoform. It’s important to note that possible read truncations and degradations are not regarded as missing sub-exons, and sub-exons smaller than 20bp do not factor into these initial decisions of excluding read-isoform mappings. When a read aligns with multiple annotated isoforms, we then align the read to sub-exons shorter than 20bps using the same coverage thresholds to refine the selection of candidate isoforms further.
5. Identify novel isoforms: Following the aforementioned procedures, if a read cannot be mapped to any existing annotated isoform, it is either a novel read to be assigned to a novel isoform, characterized by a unique combination of sub-exons, or classified as an uncategorized read. An uncategorized read is one that either maps to non-coding regions or lacks sufficient alignment evidence for classification into novel isoforms. To identify novel isoforms, we construct a graph where each node corresponds to a novel read, and edges between nodes indicate read similarities. We limit the graph size to 1500 novel nodes for computational efficiency. Two reads are connected if there is no instance where one read covers and the other skips the same sub-exons. The edge weight is the number of sub-exons to which both reads are either aligned or unaligned. Utilizing the Louvain method, we detect read communities that likely represent the same novel isoforms. For each identified read community, we calculate the mean coverage percentage for each sub-exon across all reads within the community to determine the combination of sub-exons that likely make up the novel isoform, applying a threshold of 50%. Subsequently, all novel reads are remapped to these newly identified novel isoforms following the same alignment procedures used for annotated isoforms. For the novel reads that have not been mapped to any identified novel isoforms, we progress to the next graph of unmapped reads and iterate this procedure until no further novel isoforms can be detected. We then group novel isoforms that appear to be products of read truncations. This approach yields a read-isoform compatibility matrix, facilitating the discovery of new isoforms and enhancing accuracy by minimizing false positives through careful consideration of read truncations and mismatches.
6. Generate count matrix: In the process of generating the count matrix, for reads that map to multiple annotated isoforms, we leverage the unique mapping reads at the pseudo-bulk level to infer the probability of mapping to these reads. This inference allows us to determine the read-isoform mapping by sampling from a multinomial distribution. If a read maps to what could be a novel isoform, and this mapping is supported by more than 10 reads at the pseudo-bulk level, we classify this as a novel isoform. Conversely, if there is insufficient support (fewer than 10 supporting reads), the mapping is considered to correspond to a non-categorized isoform. Utilizing cell barcode, we can then generate gene and transcript count matrices.
7. Transcriptome analysis: SCOTCH can perform transcriptome analysis by testing differential transcript usage on both gene and transcript levels and identify isoform switching events. For gene with *g* with *K* isoforms, we model the transcript expression level *X*_*ck*_ for isoform *k* in cell *c* using a Dirichlet-multinomial hierarchical model to account for transcript usage variations among cells. We assume *X*_*ck*_ ~ *MultiNomial* (*n*_*c*_*, π*_*c*_), and *π*_*c*_ *~ Dirichiet* (*α*). Here, $n_{c}=\sum_{i=1}^{K}X_{k}$ is the total transcript counts for the gene in the cell. *π*_*c*_ = (*π*_*c*,1_, … , *π*_*c,k*_) is cell-specific transcript usage. *α *= (*α*_*1*_*, … ,α*_*K*_) are the concentration parameters of the Dirichlet distribution we will estimate, and the average transcript usage for the cell population is $\bar{\pi }=\left({\bar{\pi }}_{1},\ldots ,{\bar{\pi }}_{K}\right)$ with ${\bar{\pi }}_{k}=\frac{\alpha_{k}}{\sum_{i=1}^{K}\alpha_{i}}$. Similar with Longcell, we define $\phi =\frac{1}{1+\sum_{i=1}^{K}\alpha_{i}}$ as a mean-invariant over-dispersion parameter, representing inter-cell heterogeneity. A small *ϕ* suggests that cells are likely to express various isoforms with similar usage proportions across the cell population, and a large *ϕ* value indicates a more exclusive expression manner, with each cell predominantly expressing one isoform while different cells may express others. Our goal is to estimate $\bar{\pi }$ using maximum likelihood estimation and compare between different cell populations *A* and *B* using likelihood ratio test. On the gene level, we test $H_{0}:{\bar{\pi }}_{k}^{A}={\bar{\pi }}_{k}^{B}$ for *k* = 1,…,*K* vs $H_{1}:{\bar{\pi }}_{k}^{A}\neq {\bar{\pi }}_{k}^{B}$ (for any 1 ≤ *k* ≤ *K*). On the transcript level, we test $H_{0}:{\bar{\pi }}_{k}^{A}={\bar{\pi }}_{k}^{B}$ vs $H_{1}:{\bar{\pi }}_{k}^{A}\neq {\bar{\pi }}_{k}^{B}$ for isoform *k*. Note that, we aggregate rare isoforms into one isoform type if ∑_*c*_ *X*_*ck*_ ≤ 0.05∑_*c*_ *n*_*c*_, for 1 ≤ *k* ≤ *K*, and only analyze genes with over 20 reads mapped to any known or novel isoforms. We define isoform switching events if the dominant isoform except the aggregated rare isoforms is different between two cell populations inferred by ${\bar{\pi }}^{A}$ and ${\bar{\pi }}^{B}$. The effect size is defined as $\left|\left({\bar{\pi }}_{a}^{A}-{\bar{\pi }}_{b}^{B}\right)+\left({\bar{\pi }}_{b}^{B}-{\bar{\pi }}_{b}^{A}\right)\right|$ where isoform *a* and *b* are dominant isoforms for cell populations *A* and *B*, respectively.

## Supplementary Material

Supplement 1

## Figures and Tables

**Table 1.** Summary of human PBMC benchmarking datasets generated in the current study

Sample Name	Sequencing Platform	Flowcell	Single Cell Library
Sample 7	lllumina		10x Genomics 3’ v3
Sample 7	lllumina		Parse Biosciences
Sample 7	Nanopore	R9	10x Genomics 3’ v3
Sample 7	Nanopore	R10	10x Genomics 3’ v3
Sample 7	Nanopore	R10	Parse Biosciences
Sample 8	lllumina		10x Genomics 3’ v3
Sample 8	lllumina		Parse Biosciences
Sample 8	Nanopore	R9	10x Genomics 3’ v3
Sample 8	Nanopore	R10	10x Genomics 3’ v3
Sample 8	Nanopore	R10	Parse Biosciences

**Table 2.** Number of genes with significant differential transcript usage (DTU)

	10X - wf-single-cell			10X - SCOTCH		
	Sample 7	Sample 8	Both samples	Sample 7	Sample 8	Both samples
B cells	2237	2610	1266	3198	3693	2309
Monocytes	3228	3862	2160	4479	5477	3766
NK cells	684	1383	380	959	1883	636
CD4 T cells	549	474	179	1033	1019	468
CD8 T cells	126	93	22	475	260	121

All DTU genes were identified with a p-adj) 0.05 determined by the False Discovery Rate (FDR) method.

Data are generated by 10X or Parse libraries and preprocessed by wf-single-cell or SCOTCH pipelines, denoted by single cell libraries – preprocessing pipeline.
