# Single-Cell Omics for Transcriptome CHaracterization (SCOTCH): isoform-level characterization of gene expression through long-read single-cell RNA sequencing

**DOI:** 10.1101/2024.04.29.590597

**Published:** 2024-11-21

**Authors:** Zhuoran Xu, Hui-Qi Qu, Joe Chan, Charlly Kao, Hakon Hakonarson, Kai Wang

**Affiliations:** 1. Graduate Group in Genomics and Computational Biology, University of Pennsylvania Perelman School of Medicine, Philadelphia, PA, 19104, USA; 2. The Center for Applied Genomics, Children’s Hospital of Philadelphia, Philadelphia, Pennsylvania, 19104, USA.; 3. Department of Pediatrics, The Perelman School of Medicine, University of Pennsylvania, Philadelphia, Pennsylvania, 19104, USA.; 4. Raymond G. Perelman Center for Cellular and Molecular Therapeutics, Children’s Hospital of Philadelphia, Philadelphia, PA, 19104, USA; 5. Department of Pathology and Laboratory Medicine, University of Pennsylvania, Philadelphia, PA, 19104, USA

## Abstract

Recent development involving long-read single-cell transcriptome sequencing (lr-scRNA-Seq) represents a significant leap forward in single-cell genomics. With the recent introduction of R10 flowcells by Oxford Nanopore, we propose that previous computational methods designed to handle high sequencing error rates are less relevant, and that the traditional approach using short reads to compile "barcode space" (candidate barcode list) to de-multiplex long reads are no longer necessary. Instead, computational methods should now shift focus on harnessing the unique benefits of long reads to analyze transcriptome complexity. In this context, we introduce a comprehensive suite of computational methods named Single-Cell Omics for Transcriptome CHaracterization (SCOTCH). SCOTCH supports both Nanopore and PacBio sequencing platforms, and is compatible with single-cell library preparation protocols from both 10X Genomics and Parse Biosciences. Through a sub-exon identification strategy with dynamic thresholding and read mapping scores, SCOTCH precisely aligns reads to known isoforms and discover novel isoforms, efficiently addressing ambiguous mapping challenges commonly encountered in long-read single-cell data. Comprehensive simulations and real data analyses across multiple platforms (including 10X Genomics and Parse Bioscience, paired with Illumina or Nanopore sequencing technologies with R9 and R10 flowcells, as well as PacBio sequencing) demonstrated that SCOTCH outperforms existing methods in mapping accuracy, quantification accuracy and novel isoform detection, while also uncovering novel biological insights on transcriptome complexity at the single-cell level.

## Introduction

The development of single-cell RNA sequencing (scRNA-Seq) technology represents a transformative force in genomics, offering unprecedented insights into cellular heterogeneity and gene expression dynamics^1–3^. However, when paired with conventional short-read sequencing methods, scRNA-seq encounters limitations in fully capturing complex genomic regions and providing comprehensive view of transcriptomic diversity ^4, 5^. These limitations become particularly pronounced in the context of detecting differential isoform splicing and discovering novel isoforms, as short reads cannot cover entire transcript lengths, leading to incomplete and often ambiguous reconstructions of splice variants. In contrast, long-read sequencing technologies, such as those from PacBio and Oxford Nanopore Technologies, offer a significant advantage by spanning entire gene or transcript lengths ^6–9^. Consequently, the emergence of long-read scRNA-Seq (lr-scRNA-Seq) represents a significant leap forward, enabling isoform-level resolution of genetic analysis and offering a more nuanced view of the transcriptomic landscape within individual cells, which are key to understanding cellular function and disease mechanisms ^10–13^.

In recent years, various long-read single-cell technologies have been developed, including LR-Split-seq ^14^, Nanopore-specific adaptation of 10X Genomics Chromium^15^, FLASH-Seq ^16^, among others, with each offering unique advantages. For instance, Parse Bioscience employs a split-pool combinatorial barcoding strategy, enabling the simultaneous sequencing of multiple samples, which is particularly advantageous for analyzing special cell populations, such as neurons, hepatocytes and developing cardiomyocytes, that cannot be readily assayed directly by the 10X Genomics platform unless single-nucleus RNA sequencing (snRNA-Seq) is used. However, the emergence of long-read sequencing also introduces new challenges, notably the historically higher error rates associated with this technology. These errors, associated with older generation of flowcells, complicate the process of identifying barcodes, such as cell barcodes and unique molecular identifiers (UMIs), for de-multiplexing reads and assign them to specific cells, leading to an urgent need for the development of computational tools like LongCell ^17^ and FLAMES ^18^ to correct these inaccuracies and ensure the reliability of downstream data analysis. In many cases, paired short-read sequencing is performed alongside long-read sequencing, to “guide” the process to mitigate errors and improve barcode assignment accuracy ^19–21^.

More recently, the introduction of R10 flowcells with v14 chemistry by Oxford Nanopore has reduced the per-base sequencing error rates to approximately 1% ^22, 23^. In principle, this dramatically reduced error rate made previous computational methods that were designed to handle high sequencing error rates less relevant nowadays. Additionally, the conventional practice of using paired short-read sequencing to compile a candidate barcode list for de-multiplexing long reads may no longer be necessary. Instead, computational methods should shift their focus towards exploiting the unique advantages of long reads to analyze transcriptome complexity, such as alternative splicing, alternative promoter usage, or alternative polyadenylation sites, without relying on short reads. Although computational approaches for short-read scRNA-seq have been well developed, focusing mainly on gene count quantification and cellular population clustering, they are not directly transferable to lr-scRNA-seq data to assess isoform-specific expression levels and differential isoform usage across cell populations. Several computational tools have been developed for long-read transcriptomics, each with distinct limitations. Bambu^24^ and IsoQuant^25^, originally designed for bulk RNA-seq data, are not inherently suited for single-cell analysis. Although a single cell version of Bambu is under development and not yet available, IsoQuant can currently be adapted for single-cell transcriptomics. However, IsoQuant requires users to preprocess the data to handle read de-multiplexing, making it less user-friendly and efficient for true single-cell applications. FLAMES^18^, while specifically designed for lr-scRNA-Seq data, still depends on short-read data for barcode identification, limiting its independence as a long-read-only tool, despite its capabilities in isoform discovery and splicing analysis. Isosceles^26^, another tool designed for transcript discovery and quantification in lr-scRNA-Seq, does not accommodate the diverse read structures from different single-cell platforms. Furthermore, many of these existing methods use statistical models that compare exon inclusion levels across cell populations, limiting their ability to directly assess isoform usage differences at the transcript level—an essential aspect for fully understanding transcriptome complexity. In this context, the development of new computational methods that can fully exploit the strengths of lr-scRNA-Seq, while overcoming these limitations, is critically needed.

In this study, we generated a comprehensive set of benchmarking datasets using both Illumina short-read and Oxford Nanopore long-read sequencing, encompassing different flow cells versions (R9 and R10) and single cell preparation protocols from 10X Genomics and Parse Biosciences. Our examination of lr-scRNA-seq data has shown that recent technological advancements have eliminated the need for parallel short-read sequencing, rendering previous concerns about high error rates less relevant. To address the unique challenges of lr-scRNA-seq, we introduce Single-Cell Omics for Transcriptome CHaracterization (SCOTCH), a suite of computational pipeline and statistical framework specifically designed for lr-scRNA-seq data analysis. SCOTCH is compatible with single-cell libraries from 10X Genomics and Parse Biosciences, as well as sequencing platforms from Nanopore and PacBio. It excels in detecting transcript usage differences between cell populations, identifying isoform switching events, and discovering novel isoforms. Through extensive simulation studies, we demonstrate SCOTCH’s superior performance in isoform mapping, detection, and quantification, and its ability to handle complex mapping challenges, such as overlapping genes and chimeric reads. By applying SCOTCH to human PBMC and cerebral organoid datasets, we demonstrate SCOTCH’s capability to reveal novel biological insights from lr-scRNA-seq data, providing transcript-level resolution that short-read data cannot achieve. Our study highlights lr-scRNA-seq's potential to deepen our understanding of genome regulation and cellular diversity at single-cell resolution. Additionally, the datasets generated from five distinct technical approaches provide a valuable resource for further development of computational methods.

## Results

### SCOTCH workflow and experimental design

**Figure 1.**
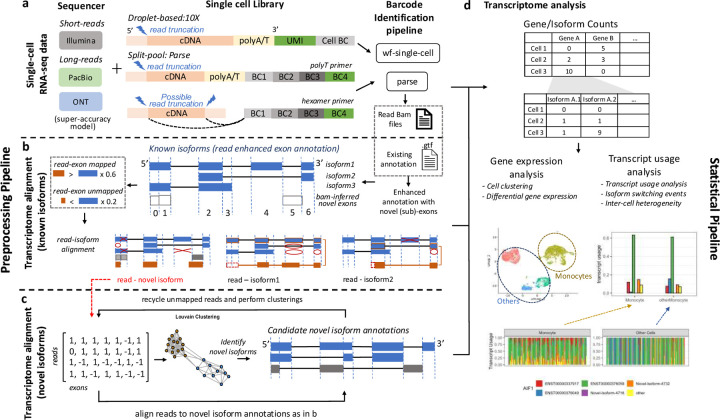
Workflow of the SCOTCH pipeline for long-read scRNA-seq analysis. **(a)** For either the 10X Genomics or Parse Biosciences single-cell library construction method, the cell barcodes and UMIs are extracted using vendor-supplied protocols. SCOTCH accounts for potential read truncations depending on primers used. **(b)** SCOTCH dissects known isoform annotations into non-overlapping exons and integrates read coverage information from BAM files to refine these annotations by identifying novel candidate exons or splitting existing exons into sub-exons. SCOTCH then maps reads to each sub-exon, applying a threshold where reads are considered mapped if it covers more than 60% of the exon’s length, and considered unmapped if it covers less than 20%. Using these read-exon mapping calls, reads are then aligned to known isoforms through a compatible matrix. **(c)** Novel reads that cannot be mapped to any known isoforms are clustered to generate novel isoform annotations at the pseudo-bulk level. Each of these novel reads is then aligned to these novel isoform annotations. **(d)** lr-scRNA-seq data generated using 10X Genomics or parse libraries can be preprocessed by vendor-supplied tools, such as the wf-single-cell or Parse pipelines for barcode identification, and then processed by SCOTCH to generate count matrices at both the gene and transcript levels. The gene-level count matrix facilitates conventional gene expression analysis, while the isoform-level count matrix can be used for transcript usage analysis through SCOTCH’s statistical pipeline.

In the current study, we introduce SCOTCH, a suite of computational pipeline and statistical framework specifically developed for analyzing lr-scRNA-seq data, as depicted in Figure 1. SCOTCH preprocessing pipeline is capable to handle data generated by both 10X Genomics and Parse Biosciences libraries, with other library generation protocols in the works, paired with Nanopore or PacBio long read sequencing. For the 10X Genomics libraries, SCOTCH detects polyA/T tails connected to UMI and cell barcodes, and account for possible read truncations at the 5’ end. For the Parse libraries, SCOTCH similarly accounts for possible 5’ read truncations if polyA/T tails can be detected in the long reads; otherwise, it considers read truncations at both 3’ and 5’ ends when hexamer primers might be used (Figure 1a). SCOTCH supports three modes of analysis: annotation-only (using only existing annotation files), annotation-free (relying solely on read coverage information), and enhanced-annotation (the default mode, combining both existing annotations and read coverage to refine annotations with novel sub-exons). This flexibility increases sensitivity for discovering novel isoforms, particularly those involving novel exons, including intron retentions and 5’/3’ alternative splicing events. SCOTCH takes BAM files with tagged barcodes generated by vendor-supplied pipelines (wf-single-cell for 10X-nanopore data, Iso-seq for 10X-PacBio data, and Parse for Parse-nanopore data) as input to align reads to known and novel isoforms, which are treated as combinations of sub-exons (Figure 1b-c). Specifically, SCOTCH encodes reads by the presence or absence of each exon, using read-exon mapping percentages. It filters out read-isoform mappings if (1) the isoform annotation includes any exons that the read skips or (2) the isoform annotation fails to encompass all exons covered by the read. For reads unmappable to any known isoforms, SCOTCH constructs novel read graphs and generates novel isoform annotations at the pseudo-bulk level using the Louvain clustering method^27^. These reads are then aligned to these newly generated isoform annotations. A read may map to multiple transcripts of a gene or, in more complex cases, ambiguously map to several overlapping genes or even nonoverlapping genes, as seen with chimeric reads. To resolve these ambiguities, SCOTCH adopts both a gene-specific dynamic thresholding strategy and the incorporation of read mapping scores (see Methods) to prioritizes the most likely transcript or gene assignment.

After preprocessing lr-scRNA-seq data, we aim to perform analysis with transcript-level resolution to unveil alterations obscured by conventional differential gene expression analysis. Differential transcript usage (DTU), which is defined as variations in relative abundance of transcripts of the same gene across different conditions or cell types, explain changes in phenotype between cell types, tissues, or disease cohorts ^28–30^. The SCOTCH statistical pipeline, which is inspired by LIQA ^31^ for truncation handling and LongCell ^17^ for exon-inclusion analysis, can be used to identify DTU on both the gene and transcript levels (Figure 1d, Methods). For each gene, SCOTCH estimates the average transcript usage of a cell population by fitting a Dirichlet-multinomial distribution, which captures inter-cell variations quantified by the over-dispersion parameter ϕ. This parameter is mean invariant, where small values indicate cells with similar isoform co-expression patterns, and large values suggest a more exclusive expression mode, where isoform usage patterns are more variable across cells. Employing likelihood ratio tests, we assess whether transcript usage differ between two cell populations at the gene level, and we examine whether a specific transcript is differentially utilized at the transcript level. One special case of transcript usage alteration is isoform switching, here defined as changes of the dominant isoform between two cell populations (we note that it may also be denoted as a synonym for DTU in some publications). SCOTCH is designed to pinpoint isoform switching events, whose effect size is measured by the absolute sum of differences in the dominant isoform proportions between the two cell populations.

To benchmark our methods and assess how SCOTCH aids in lr-scRNA-seq analysis for understanding transcriptome complexity, we first simulated lr-scRNA-seq data for two distinct cell populations with ground truth. Additionally, we generated a substantial amount of evaluation data for two human peripheral blood mononuclear cells (PBMC) samples using both 10X Genomics and Parse libraries, Illumina and nanopore sequencings (Table 1). Throughout the study, we first demonstrated that the availability of R10 flowcells facilitates isoform-level analysis without paired short reads or complex computational methods previously required to handle high sequencing error rates. This is shown through several key comparisons, including short-read versus long-read sequencing, R9 versus R10 flowcells sequencing technologies, and the 10X Genomics versus the Parse Bioscience library preparation system. Further into the study, we compared SCOTCH’s performance with several benchmark methods and analyzed lr-scRNA-seq data from PBMCs and human cerebral organoids^32^ to showcase the effectiveness of SCOTCH’s statistical framework in analyzing DTU, detecting isoform switching events, and assessing inter-cell heterogeneity.

### R10 flowcells facilitate isoform-level analysis with improved sequencing quality

When preprocessing lr-scRNA-seq data with library constructed on the 10X Genomics platform, a threshold of edit distance (ED) is set for UMI clustering and cell barcode resolution. This ED threshold determines the stringency of the error correction steps for UMIs and cell barcodes, directly impacting the precision of read allocation to particular cells and the effectiveness of molecule de-multiplexing. The conventional wisdom is that R9 flowcells need an ED of 2 for accurate read-cell mapping ^33^, owing to their higher sequencing errors compared to short-read sequencing. However, the newer R10 flowcells display improved sequencing quality, particularly in homopolymers regions ^34^, where they have lower deletion rates in thymine and adenine sequences than R9, albeit with increased mismatches ^35^. The R10’s ability to match Illumina’s sequencing quality standards yields flexibility in choosing between ED1 and ED2, allowing for more efficient and accurate read mappings to the corresponding cells.

**Figure 2.**
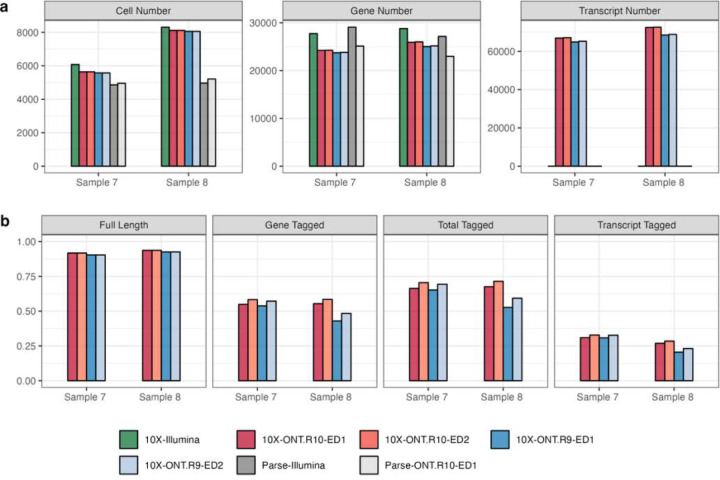
Influence of edit distance (ED) criteria on final results of five single-cell library + sequencing platforms generated by vendor computational pipeline: 10X + Illumina, Parse + Illumina, 10X + Nanopore_R9, 10X + Nanopore_R10, Parse + Nanopore R10. **(a)** The number of cells, genes and transcripts that are identified based on different flowcell versions and different ED thresholds (1 or 2). **(b)** The fraction of reads that are classified as full-length, gene-tagged, total-tagged, and transcript-tagged in two samples sequenced by both R9 and R10 flowcells on the Nanopore platform.

To empirically substantiate the technical advancements offered by R10 flowcells that facilitate isoform-level analysis, we systematically examined and compared sequencing statistics across different combinations of single-cell libraries and sequencing technologies (10X + Illumina, Parse + Illumina, 10X + Nanopore_R9, 10X + Nanopore_R10, Parse + Nanopore_R10) that are preprocessed by vendor-supplied computational pipelines (Table S1, Figure 2). Regardless of whether an ED of 1 or 2 is applied, R10 flowcells align more closely with short-read sequencing in terms of median numbers of genes and UMIs per cell (Table S1), as well as the total counts of cells, genes, and transcripts (Figure 2a). R10 flowcells also show higher proportions of reads categorized as full length, total-tagged, gene-tagged, and transcript-tagged (Figure 2b), indicating higher sequencing quality. When applying an ED of 2, both R9 and R10 flowcells display an increase in sequencing saturation and elevated median gene, transcript, and UMI counts per cell compared to using ED of 1. This is because a higher ED threshold leads to more lenient clustering, thereby enabling the recognition of more unique transcripts. Notably, the increase seen with ED2 relative to ED1 is less pronounced for R10 than R9, and both ED1 and ED2 exhibit comparable performance in terms of the total numbers of cells, genes, and transcripts for R10 (Figure 2a, Figure S1), suggesting that the improved sequencing accuracy of R10 flowcells reduces the need for a higher ED for reliable transcript identification. Additionally, R10 consistently outperforms R9 in sequencing saturation, and median numbers of genes, transcripts, and UMI counts per cell across both ED settings, affirming its ability to capture a more comprehensive transcriptome profile even at a stricter ED threshold.

### SCOTCH accurately characterizes and quantifies transcripts in simulation studies

To comprehensively evaluate SCOTCH performance in characterizing and quantifying transcripts, as well as supporting downstream transcriptome analysis, we simulated ground-truth nanopore scRNA-seq data for two distinct cell populations using a modified version of lrgasp-simulation specifically adapted for single-cell data (see Methods). Specifically, we simulated reads from 1080 expressed genes on chromosome 6, with each having at least two gene isoforms based on GENCODE gene annotation^36^. To examine the ability of SCOTCH in the discovery and annotation of novel isoforms, we randomly removed 2466 (30%) isoforms, designating them as ground-truth “novel” isoforms, while the remaining 5762 (70%) isoforms in all 1080 genes were used as the existing annotation file for all tools. To assess whether SCOTCH’s gene- and transcript-level count matrices preserved underlying differential transcript usage signals relevant to disease studies, we set the transcript compositions of 540 (50%) genes to differ between two cell populations, with the remaining 540 genes maintaining the same transcript compositions across both groups. In total, we simulated 15,381,985 reads for two cell populations, each having 1000 cells. We compared performance of SCOTCH (default enhanced-annotation mode), SCOTCH.ao (annotation-only mode), and several other methods including IsoQuant, Bambu, FLAMES (modified by us to process single-cell data), and Isosceles (with both strict and loose modes).

**Figure 3.**
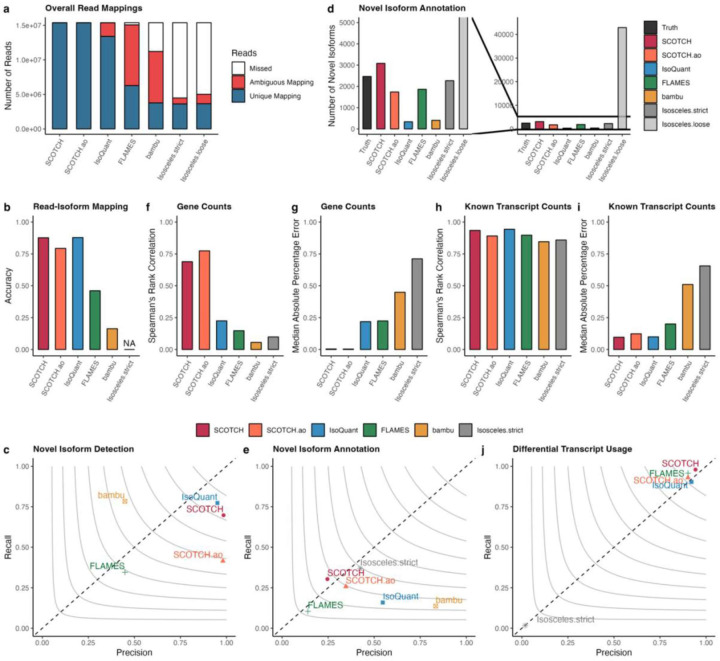
Simulation study to evaluation SCOTCH performance compared with benchmark methods. **(a)** Bar plots showing number of reads that mapped to one isoform uniquely (unique mapping), multiple isoforms ambiguously (ambiguous mapping), and missed by tools. **(b)** Accuracy of read-isoform mappings including known and novel transcripts. Since Isosceles does not output read-isoform mapping information, NA was annotated. **(c)** Precision and recall for classifying reads to known or novel isoforms. **(d)** Number of novel isoforms annotated by different tools. The left panel shows zoomed in view. **(e)** Precision and recall for annotating novel isoforms. **(f)** Spearman’s rank correlation of gene expressions between ground-truth values and tools’ output for 1080 genes on the bulk level. **(g)** Median absolute percentage error of gene expressions between ground-truth values and tools’ output for 1080 genes on the bulk level. **(h)** Spearman’s rank correlation of transcript expressions between ground-truth values and tools’ output for 5762 known transcripts on the bulk level. **(i)** Median absolute percentage error of transcript expressions between ground-truth values and tools’ output for 5762 transcripts on the bulk level. **(j)** Precision and recall for identifying genes with differential transcript usage (adjusted-p value < 0.01). **(d-e, j)** contour lines represent F1 scores.

We first examined the composition of mapped reads for each method. As shown in Figure 3a, most competing methods display a significant proportion of ambiguously mapped reads, in some cases even surpassing the number of unique mappings and exhibit a notable number of missing reads. This is a known challenge in long-read single-cell data, and it has influences on downstream data analysis since a large proportion of reads cannot be assigned to transcripts. In contrast, SCOTCH and SCOTCH.ao achieve the highest number of uniquely mapped reads with minimal ambiguity, showcasing a unique advantage of handling ambiguous mapping. We then compared read-isoform mapping accuracy for both known and novel isoforms. Figure 3b shows that SCOTCH, in enhanced-annotation mode, achieves accuracy of 0.877, comparable to IsoQuant (0.878). The annotation-only mode (SCOTCH.ao) also performs well with an accuracy of 0.793, significantly outperforming other tools such as FLAMES (0.461) and Bambu (0.163). The accuracy difference between SCOTCH and SCOTCH.ao likely stems from SCOTCH’s ability to refine sub-exon annotations using read coverage information, enhancing its ability in novel isoform detection. To further explore this, we treated read-isoform mappings as a classification task for identifying novel isoforms. As seen in Figure 3c, SCOTCH accurately detects novel isoforms, with an F1 score (0.817) slightly lower than IsoQuant (0.851). SCOTCH and SCOTCH.ao exhibit similarly high precision (SCOTCH: 0.984, SCOTCH.ao: 0.981), but SCOTCH demonstrates significantly higher recall (0.698) than SCOTCH.ao (0.415), indicating its enhanced sensitivity in detecting novel isoforms. In comparison, Bambu and FLAMES show much lower precision and recall. Isosceles does not report read-isoform mappings and was thus excluded from these comparisons. We then evaluated the performance of various tools in annotating identified novel isoforms. Isosceles.strict, SCOTCH, SCOTCH.ao, and FLAMES identified 2268, 3084, 1739, and 1864 novel isoforms, respectively, which are relatively close to the ground truth of 2466. In contrast, IsoQuant (717) and Bambu (404) significantly underestimate the novel isoform number, while Isosceles.loose (42,867) vastly overestimates it (Figure 3d). We then used the strict mode for Isosceles in all further comparisons. Next, we used gffcompare^37^ to assess the accuracy of novel isoform annotations. We found Isosceles.strict achieves the highest F1 score (0.395), followed by SCOTCH.ao (0.295), SCOTCH (0.272), and IsoQuant (0.246), respectively (Figure 3e). After evaluating transcript characterization, we shifted our focus to quantification, assessing gene (Figure 3f-g) and transcript counts (Figure 3h-i). SCOTCH and IsoQuant exhibit similarly high correlations with ground truth (SCOTCH: 0.926, IsoQuant: 0.943), and low MAPE values (SCOTCH: 0.096, IsoQuant: 0.099) for known transcripts. While other tools perform worse in transcript quantification, the differences are not as pronounced. However, for gene-level quantifications, SCOTCH and SCOTCH.ao significantly outperform other tools, achieving correlation coefficients of 0.689 and 0.774, respectively, both with remarkably low MAPE values (SCOTCH: 0.004, SCOTCH.ao 0.003). In comparison, IsoQuant only achieved a correlation of 0.225 and a MAPE of 0.218, with other tools performing even worse. This superior performance can be attributed to SCOTCH’s ability to resolve ambiguous mappings more effectively, as seen in Figure 3a, where SCOTCH showed a higher proportion of uniquely mapped reads with minimal ambiguity. We finally assessed how each tool aids in transcriptome analysis by preserving the underlying differential transcript usage (DTU) signals (Figure 3j). SCOTCH, SCOTCH.ao, FLAMES and IsoQuant closely align with the ground truth in terms of precision and recall, with SCOTCH outperforming the others. However, Isosceles.strict performs the worst, with near-zero precision and recall, likely due to the vast number of missed and ambiguous reads (Figure 3a). These results demonstrate SCOTCH’s effectiveness in accurately capturing DTU signals, further supporting its robustness in transcript-level analysis.

### Consistency and reproducibility between different sequencing technologies, single-cell libraries, and computational pipelines

**Figure 4.**
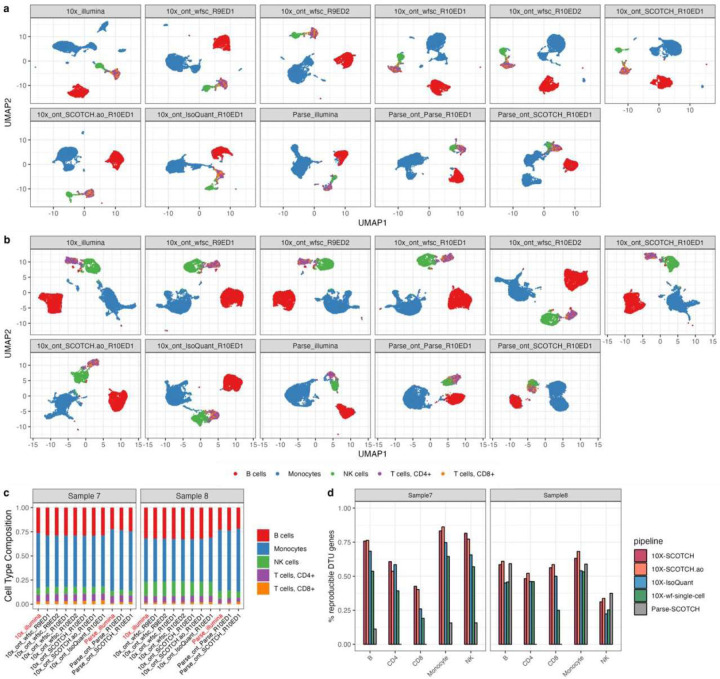
Consistency and reproducibility analysis comparing various technical platforms and pipelines. **(a-b)** UMAP visualization for clustering of different cell types comparing between 10X Genomics and Parse single-cell libraries, short-read and long-read scRNA-seq, R9 and R10 flowcells, Edit Distance of 1 and 2, as well as vendor (wf-single-cell, wfsc), SCOTCH pipelines, and IsoQuant on the same PBMC samples. Panel names are in the format of single-cell libraries_sequencing technology_computational pipeline_flowcell and edit distance. **(a)** PBMC sample 7. **(b)** PBMC sample 8. **(c)** Cell type compositions for sample 7 in a and sample 8 in b. **(d)** Percentage of DTU genes that are also identified in the other sample analyzed by SCOTCH and wf-single-cell pipelines.

After the simulation studies, we applied SCOTCH, SCOTCH.ao, IsoQuant, and the wf-single-cell pipeline to two human PBMC samples processed with different single-cell libraries and sequencing protocols. Due to Isosceles.stricťs high rate of missed reads, FLAMES' reliance on short-read barcoding, Bambu's focus on bulk sequencing, and Parse’s inability to generate transcript-level matrices (as of November 2024), these tools were excluded from this analysis. We first compared cell type clusters on the gene level across short-read and long-read scRNA-seq, 10X Genomics and Parse single-cell libraries, R9 and R10 flowcells, and with Edit Distance of 1 and 2, as well as different computational pipelines using PBMC samples (Figure 4a-c). The cell type clusters were generally consistent in UMAP visualizations and cell type proportions across different platforms and analytical methods, with monocytes being the most prevalent cell type, followed by B cells. These patterns were reproducible across two samples, underscoring the reliability of cell type identification across diverse sequencing technologies, library preparations, and analytical approaches on the gene level. On the transcript level, we assessed the number of DTU genes across two samples identified by different computational methods, utilizing data generated by 10X Genomics library with nanopore R10 flowcells (ED of 1). As shown in Table 2, the SCOTCH and SCOTCH.ao consistently identified a greater number of DTU genes across different cell types than the wf-single-cell pipeline for most cell types, with IsoQuant detecting significantly fewer DTU genes. Moreover, the results from the second sample confirmed the superior reproducibility of SCOTCH and SCOTCH.ao, with both methods maintaining a higher percentage of reproducible DTU genes across samples compared to other tools (Figure 4d). Owing to the low sequencing depth of the Parse library, we proceeded to determine whether the Parse platform enables the identification of key DTU genes that were also detected using the 10X Genomics platform. We found that over half of DTU genes detected in B, monocytes, and NK cells by Parse-SCOTCH were validated by 10X-SCOTCH (Figure S3), suggesting strong consensus between both single-cell library construction methods.

### SCOTCH aids in revealing transcriptome insights beyond gene expression

**Figure 5.**
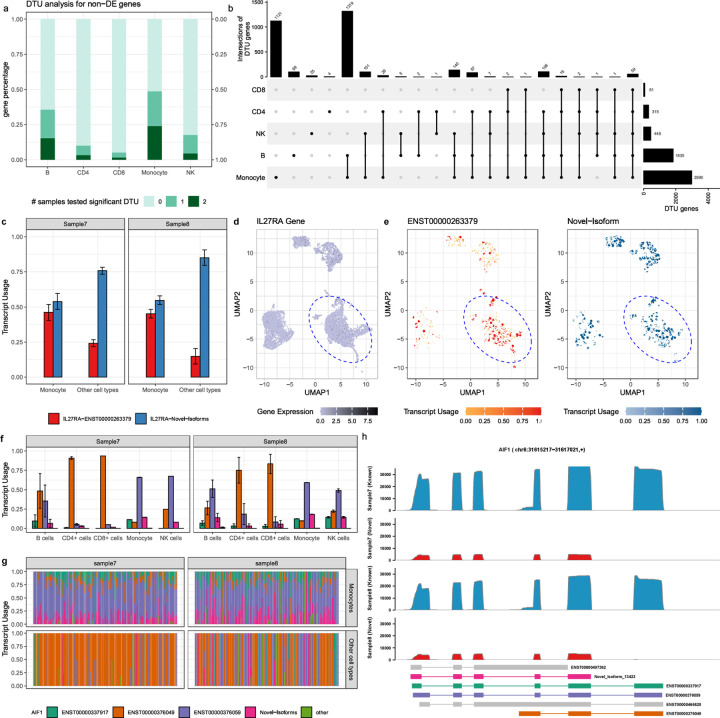
Transcript usage analysis for two PBMC samples using SCOTCH. **(a)** Proportions of non-differentially expressed (DE) genes with differential transcript usage (DTU) in various cell types. The darkness of colors indicates the number of samples in which significant DTU was observed. **(b)** Upset plot displaying the intersections of significant DTU genes of each cell type in against others in both sample 7 and sample 8. **(c)** Bar plot displaying the estimated average transcript usage for *IL27RA* gene isoforms comparing monocytes and other cell types for sample 7 and sample 8. **(d)** UMAP visualization for expression levels of the *IL27RA* gene. **(e)** UMAP visualization for *IL27RA* gene isoform usages of each individual cell. Cells within blue circles are monocytes. **(f)** Bar plot displaying estimated average transcript usages for *AIF1* gene isoforms comparing monocytes and other cell types for sample 7 and sample 8. **(g)** Distribution of *AIF1* gene transcript usage of monocytes and all other cell types for sample 7 and sample 8. Each column represents a cell, with colors indicating isoform compositions used within each cell. Monocytes are downsampled to the same number of other cell types. **(h)** Displayed are reads mapped to known *AIF1* isoforms (ENST0000376049, ENST00000376059, ENST00000337917) and a novel isoform (novel isoform-13423) for sample 7 and sample 8. The identified novel isoform does not contain the highlighted exon on the 3’ end. Error bars show standard deviations. Thresholds for non-DE genes are p-adj > 0.05, thresholds for DTU genes are p-adj < 0.01.

After confirming SCOTCH’s consistency and reproducibility across different platforms, we evaluated its ability to uncover transcript-level changes which conventional gene-level analysis may miss. To do this, we performed differential gene expression (DGE) analysis and differential transcript usage (DTU) analysis by comparing each cell type against the others using the gene-level, and transcript-level count matrices, respectively. We found that 1% to 24% of the genes, which were not differentially expressed between cell populations, exhibited DTU in both samples, while 5% to 49% exhibited DTU in at least one sample (Figure 5a). These findings suggest that relying solely on gene expression comparisons could overlook significant biological signals introduced by transcript variations. Figure 5b displays the number of DTU genes identified in both samples, highlighting differential transcript usage as a common feature among various cell types. Monocytes exhibited the highest number of DTU genes (n = 2990), with 1121 genes being unique to this cell type, suggesting that distinct isoforms could play roles specific to monocyte functions. B cells had the second-highest DTU count (n = 1835), with 1728 genes overlapping with monocytes, indicating shared transcriptomic characteristics and pathways (Figure S4). NK cells, along with CD4, and CD8 T cells had a lower number of DTU genes, likely due to their lower prevalence in the samples. These findings underscore the cell-type-specific nature of transcript usage. To further explore these patterns, we conducted gene set enrichment analysis to identify biological processes related to cell type-specific transcript usage (Figure S4). We observed that certain biological processes are consistently enriched in most cell types, such as ribosome and antigen processing and presentation. Monocytes and B cells show the most similar pathway enrichment patterns, reflecting the substantial overlap of DTU genes between them.

To showcase the utility of SCOTCH in identifying DTU genes, especially when overall gene expression levels remain unchanged, we examined the *IL27RA* gene, which encodes a component of the receptor for interleukin-27 (IL-27) and plays critical roles in immune cell differentiation and the control of cytokine production^38^. The exploration of *IL27RA* isoforms has therapeutic relevance, as a soluble form of IL-27Rα has been shown to act as a natural antagonist to IL-27^39^. Manipulating the expression of various isoforms could provide novel methods to modulate immune responses in diseases influenced by IL-27. As depicted in Figure 5c-e, transcript usage for *IL27RA* significantly differs on both the gene level (p-adj < 0.0001) and the transcript level for ENST00000263379 (p-adj < 0.0001), and the novel-isoform (p-adj < 0.0001) between monocytes and other cell types in both sample 7 and sample 8. In particular, transcript usages for ENST00000263379 and the novel isoform are nearly equivalent in monocytes for two samples, whereas in other cell types, the novel isoform is the predominant isoform (sample7: 0.76 ± 0.03, sample8: 0.85 ± 0.06). Despite these variations in transcript usage, overall *IL27RA* expression levels remain unchanged between monocytes and other cell types, with a p-adj value of 1 (Figure 5d). These results suggest SCOTCH’s ability to uncover transcriptomic variations and identify novel isoforms, even when gene expression levels show no significant changes.

We further examined the *AIF1* gene as another case study to demonstrate the utility of SCOTCH in DTU analysis. *AIF1* is instrumental in cytoskeletal rearrangements and cell migration^40^. In immune responses, *AIF1* acts as a critical modulator of inflammation and immune cell activation^41^. *AIF1* isoforms may vary in their expression patterns, subcellular localization, or interaction partners, influencing the roles of *AIF1* in various cellular processes, including inflammation and immune responses^42^. Figure 5f illustrates the DTU patterns for *AIF1* in two samples, comparing each cell type to others. ENST00000376059 is the predominant isoform in monocytes and NK cells, whereas ENST00000376049 dominates in CD4+ and CD8+ T cells. In addition to assessing transcript usage differences, SCOTCH elucidates isoform co-expression patterns using the mean-invariant over-dispersion parameter ϕ. To account for the imbalance in the numbers of cells expression *AIF1* isoforms, we down-sampled monocyte data to match the number of cells from other types, enabling a visually comparable analysis of cell type-specific isoform co-expression. Figure 5g shows that monocytes typically express multiple isoforms simultaneously with similar usage proportions (Sample7:ϕ=0.010, Sample8:ϕ=0.008). In contrast, other cells exhibit greater heterogeneity, often exclusively expressing a single isoform, as indicated by higher dispersion values (Sample7ϕ=0.394, Sample8:ϕ=0.410). These diverse isoform usage patterns have implications for the intricate roles of *AIF1* in cellular and immune processes. Although *AIF1*’s transcript usage was found to be significant based on count matrices generated by both wf-single-cell pipeline and IsoQuant for monocytes, neither pipeline identified any novel isoforms. IsoQuant, as shown in simulation studies, lacked the sensitivity to detect novel isoforms, while the wf-single-cell pipeline was not designed to identify them. In contrast, SCOTCH identified a novel isoform for *AIF1*. This novel isoform omits the final exon found in the known isoforms of ENST00000376049, ENST00000376059, and ENST00000337917 (Figure 5h). Instead, it directly connects to the polyadenylation site (polyA tail), producing transcripts with alternative terminal sequences. SCOTCH’s capability to uncover these novel isoform structures underscore its potential to yield deeper insights into transcriptomic complexity.

### SCOTCH uncovers cell-type-specific transcript usage in human cerebral organoids

**Figure 6.**
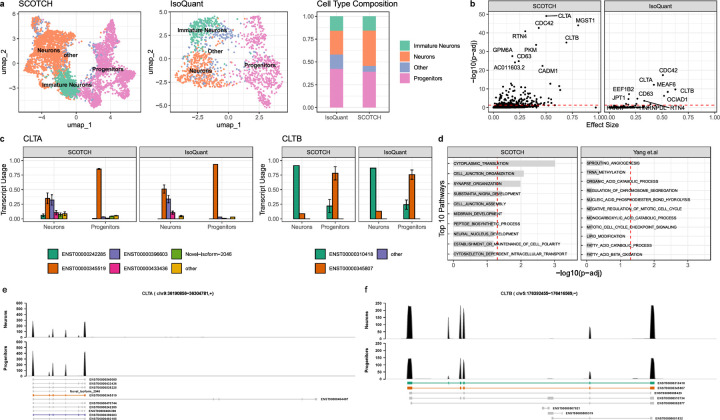
Transcript usage analysis on human cerebral organoid samples. **(a)**. Displayed are UMAP visualization for cell type clusters generated by SCOTCH (left) and IsoQuant (middle), and corresponding cell type proportions (right). **(b)** Scatter plot showing DTU genes between neurons and progenitors detected by SCOTCH (left) and IsoQuant (right). The x-axis represents the effect size, defined as the maximum transcript usage difference between two cell types for each gene. The y-axis represents negative log10-transformed adjusted p-values. Top 10 significant DTU genes are labeled. **(c)** Bar plots showing the estimated average transcript usage for genes *CLTA* (left) and *CLTB* (right) in neurons and progenitors. **(d)** Top 10 enriched pathways for DTU genes identified by SCOTCH (left) and differential spliced genes identified by Yang et al^32^ (right). The x-axis represents negative log10-transformed adjusted p-values, with red dotted lines indicating the significance threshold at an adjusted p-value of 0.05. **(e)** Displayed are reads mapped to *CLTA* genes in neurons and progenitors. **(f)** Displayed are reads mapped to *CLTB* genes in neurons and progenitors. **(e-f)** The most frequently used transcripts are colored in the annotation panel. The red-highlighted region marks exons with different splicing patterns between two isoforms. Error bars show standard deviations. Thresholds for DTU genes are p-adj < 0.05.

To investigate SCOTCH’s capability to uncover cell-type-specific transcript usage on the PacBio sequencing platform, we applied SCOTCH and IsoQuant to seven human cerebral organoid samples sequenced with the 10x-PacBio protocol^32^. Interestingly, we observed that the gene-level count matrix generated by IsoQuant is much sparser than that produced by SCOTCH (Table S3). Consequently, after preprocessing, SCOTCH retained a larger number of cells (n=4664) compared to IsoQuant (n=1406), enabling more accurate cell type annotations. As shown in Figure 6a, both methods produced similar proportions of progenitors and immature neurons. SCOTCH achieved a balanced proportion of neurons and progenitors, consistent with the findings from the original study. In contrast, IsoQuant’s sparse count matrix led to weak gene signature enrichment, making some clusters difficult to annotate. As a result, these clusters were labeled as “Other” cell types due to insufficient gene signature specificity. When comparing transcript usage between neurons and progenitors, SCOTCH identified 194 DTU genes, while IsoQuant detected only 13 (Figure 6b), highlighting SCOTCH’s superior sensitivity in capturing cell-type-specific transcriptomic differences. The 194 DTU genes identified by SCOTCH are enriched in pathways related to cytoplasmic translation, cell junction organization, synapse organization, and substantia nigra development (Figure 6d). These genes are also enriched for postsynaptic density (PSD) proteins^43^ and targets of FMRP^44^ (Figure S5). Among the DTU genes, SCOTCH and IsoQuant both identified *CLTA* and *CLTB* as top 10 candidates, displaying consistent transcript usage patterns between the methods (Figure 6c). In progenitors, *CLTA* is predominantly expressed through the isoform ENST00000345519, whereas neurons exhibit a balanced usage of isoforms ENST00000396603 (neurons vs progenitors: SCOTCH: 0.323±0.089 vs 0.031±0.002, adjusted p-value < 0.0001; IsoQuant: 0.338 ± 0.063 vs 0.030 ± 0.000, adjusted p-value < 0.0001) and ENST00000345519 (neurons vs progenitors: SCOTCH: 0.350±0.093 vs 0.853±0.009, adjusted p-value < 0.0001; IsoQuant: 0.510±0.071 vs 0.933±0.000, adjusted p-value < 0.0001). For *CLTB*, neurons primarily express the isoform ENST00000310418 (neurons vs progenitors: SCOTCH: 0.913±0.000 vs 0.220±0.113, adjusted p-value < 0.0001; IsoQuant: 0.870±0.000 vs 0.243±0.080, adjusted p-value < 0.0001), while progenitors shift to isoform ENST00000345807 (neurons vs progenitors: SCOTCH: 0.087 ±0.000 vs 0.780 ±0.113, adjusted p-value < 0.0001; IsoQuant: 0.130±0.000 vs 0.757±0.080, adjusted p-value < 0.0001). The isoform usage patterns can also be observed in the read coverage plots (Figure 6e-f), where neurons and progenitors display distinct coverage profiles across specific exons for *CLTA* and *CLTB*. These DTU patterns are consistent with previous studies, where neuron-specific splicing of *CLTA* and *CLTB* influences synaptic vesicle cycling, neural differentiation, and brain development^11, 45–48^.

## Discussion

In this study, we generated a benchmarking dataset of two human PBMC samples and demonstrated the improved read quality of R10 flowcells over R9 flowcells through comparing five different sequencing approaches: 10X + Illumina, 10X + ONT_R9, 10X + ONT_R10, Parse + Illumina, and Parse + ONT_R10, under ED settings of 1 and 2 (Table 1). These advancements with R10 flowcells underscore the technical potential of long-read single-cell RNA sequencing (lr-scRNA-seq), enabling a shift in computational tool development from barcode correction to better unraveling transcriptome complexity. In response, we introduced SCOTCH, a suite of computational and statistical pipelines specifically designed for the processing and analysis of lr-scRNA-seq data. SCOTCH’s preprocessing pipeline is compatible with both Nanopore and PacBio sequencing platforms and supports single-cell libraries from 10X Genomics and Parse Biosciences. Notably, the Parse Biosciences platform offers a unique advantage by enabling simultaneous sequencing of multiple samples, eliminating the need for additional data integration and facilitating the analysis of specialized cell populations.

SCOTCH incorporates several innovative design elements that make it highly effective for analyzing lr-scRNA-seq data. First, SCOTCH generates read-isoform mappings by aligning reads to non-overlapping sub-exons, whose combinations define distinct isoforms. To improve accuracy, SCOTCH integrates read coverage data to refine sub-exon annotations, allowing for the detection of a wide range of novel isoforms, including those with intron retention and alternative transcription start and end sites. In contrast, SCOTCH.ao relies solely on existing annotations, limiting its ability to identify novel isoforms that involve new exon structures. Second, SCOTCH employs a clustering approach to annotate and identify novel isoforms at the pseudo-bulk level, making it more resilient to noise, including read truncation, than other methods. Third, SCOTCH effectively addresses complex read-mapping challenges by using a dynamic thresholding strategy and mapping scores to resolve ambiguities when reads map to multiple isoforms within a gene, overlapping genes, or multiple non-overlapping genes. Additionally, SCOTCH enables the simultaneous preprocessing of multiple samples within a study, producing a consensus annotation file tailored to the experimental design. Finally, SCOTCH’s isoform-based analyses provide clearer biological interpretation and have shown superior performance over exon-based approaches in detecting isoform-level variations.^45^ Together, these features establish SCOTCH as a robust and adaptable tool for transcriptome analysis in lr-scRNA-seq.

We also acknowledge several limitations inherent to the SCOTCH pipeline and our study. First, SCOTCH currently performs Louvain clustering on reads that cannot be mapped to any known isoforms of a gene and uses read clusters to generate candidate novel isoform annotations. While effective, this approach could be further refined by weighting reads based on alignment quality—an approach inspired by methods like LIQA ^31^—to improve the accuracy of novel isoform annotations by accounting for variations in read quality. Second, SCOTCH’s statistical framework currently performs one-way comparisons, focusing on mean shifts in transcript usage composition vectors between two cell populations. Extending this to Dirichlet regression would allow SCOTCH to incorporate additional covariates, such as batch effects, in complex study designs. This enhancement would enable simultaneous testing of both mean shifts and variance changes in transcript usage, making SCOTCH more adaptable to nuanced experimental setups. Moreover, a set of five samples was pooled by the Parse single-cell library construction approach and subsequently sequenced using R10 flowcells, yet only two of them are used in the current study. Given the relatively low sequencing depth per sample, direct comparisons between the Parse results and those from the 10X Genomics library cannot be completed in the current study. However, we have demonstrated consistency in the detection of differentially used transcripts (DTUs) when there is an adequate number of cells and sufficient coverage.

In the rapidly advancing field of single-cell and single-nucleus sequencing, current protocols and computational methodologies present several challenges but also offer significant opportunities for improvement. One major challenge is the analysis of full-length mRNA isoforms, which is essential for accurately profiling cellular subtypes and identifying transcript variants, such as alternative splicing and alternative polyadenylation sites ^5^. Emerging tools like SCOTCH, alongside improvements in vendor-supplied computational pipelines, are paving the way for deeper insights into complex gene expression patterns. Additionally, high dropout rates significantly impede the accuracy of gene expression profiling ^49^, an issue that is particularly pronounced in long-read transcriptome analysis at the transcript level. A promising future direction involves the development of computational methods specifically tailored for long-read data to mitigate this issue, building on approaches that have shown effectiveness in short-read data^50–52^. Furthermore, non-polyA RNA, which includes various functionally important regulatory RNAs ^53^, is better captured by the Parse Biosciences protocol due to its use of a mix of polyA primers and random hexamers, unlike the 10X Genomics protocol that requires a polyA tail exclusively. Future experimental approaches could further refine these techniques to provide a more comprehensive analysis of the transcriptome, ensuring that even non-polyadenylated RNA molecules are accurately represented. Additionally, future improvements are poised to refine unsupervised clustering utilizing full-length transcript information, leading to enhanced resolution at the transcript level and deeper biological insights. Another exciting frontier is the assessment of RNA velocity through long-read sequencing data ^54^, which holds the potential to drastically alter our understanding of RNA dynamics and regulatory mechanisms. Lastly, advancements in sequencing technology that increase depth and reduce costs are expected to facilitate more precise allele-and isoform-resolution counting, thereby broadening the applicability of transcriptomic analyses across diverse cell populations. This progression promises a more detailed and nuanced understanding of transcriptomics in health and disease. Collectively, these efforts are critical for refining lr-scRNA-seq analyses and advancing comprehensive cellular profiling.

In summary, by capturing the repertoire of transcriptional isoforms across diverse cell types, SCOTCH provides a foundation for delving into the functional significance of these isoforms in various cell types and holds promise in unraveling the complexity of cellular processes.

## Methods

### Preparation of the PBMC samples

Blood samples from two de-identified individuals, one male and one female, were collected using tubes coated with EDTA. These samples were promptly processed to separate PBMCs through Ficoll density gradient centrifugation at the Center for Applied Genomics (CAG) at the Children's Hospital of Philadelphia (CHOP). The Institutional Review Board at the CHOP approved this study.

### Single cell library preparation on the 10X Genomics platform

RNA samples were processed using 10X Genomics Next GEM Single Cell 3' Kit (V3.1) following manufacturer recommended protocols. During 10x library preparation, an aliquot of about 10ng of full-length amplified barcoded cDNA was taken for Nanopore sequencing. The remaining amount was processed further using the standard 10X protocol for Illumina sequencing.

### Single cell library preparation on the Parse Biosciences platform

RNA samples were processed using Parse Bioscience Evercode WT Mini v2 Kit following the manufacturer recommended protocol. During the first barcoding step, two control samples were distributed across 3 wells, Sample 7 and Sample 8 were distributed across 4 wells each, and a PBMC control was added to the last well. Approximately 70ng of each sublibrary was taken for Nanopore sequencing while remaining amount was used for Illumina sequencing.

### Oxford Nanopore sequencing of single-cell libraries

The single cell cDNA was processed for Nanopore sequencing using the Oxford Nanopore Technology (ONT) Ligation Sequencing Kit V14 (ONT, SQK-LSK114), cDNA-PCR Sequencing Kit (ONT, SQK-PCS111), or a combination of the cDNA-PCR Sequencing Kit and the Rapid Sequencing Kit (ONT, SQK-RAD114), and two custom primers, 5'-/5Biosg/CAGCACTTGCCTGTCGCTCTATCTTCCTACACGACGCTCTTCCGATCT-3' and 5'-CAGCTTTCTGTTGGTGCTGATATTGCAAGCAGTGGTATCAA CGCAGAG-3’. The primers target the Illumina Read 1 sequence and the 10X Genomics template switch oligo sequence. 10ng of the single cell cDNA was mixed with 10 uM of both custom primers and LongAmp Hot Start Taq 2X Master Mix (NEB, M0533S). Amplification in a thermal cycler was performed using the following condition: 94C for 3 minutes, four cycles of 94C for 30 seconds, 66C down to 58C for 40 seconds, 58C for 50 seconds, and 65C for 6 minutes, a final extension cycle of 65C for 10 minutes, and a hold of 4C. Clean up was performed using AMPure XP beads (Beckman Coulter^™^ cat # A63881) with a 1.25x solution to beads ratio, and washed twice with freshly made 200 ul of 80% ethanol, and the amplified cDNA was eluted with 10 ul of nuclease-free water.

Full-length cDNA was isolated using M280 streptavidin, 10 ug/ul (Invitrogen, 11205D), which the biotinylated cDNA binds to. 4 ml of a 2X wash/bind buffer was prepared with 10 mM Tris-HCl pH 7.5, 2 M NaCl, and 1 mM EDTA. Half of the 2X wash/bind buffer was used to make a 1X wash/bind buffer to us as a wash buffer. A 5 ug/ul streptavidin bead was made by replacing the buffer from 5 ul of the streptavidin beads with 10 ul of the 2X wash/bind buffer. 10 ul of the biotinylated cDNA was added to the 5 ug/ul beads and incubated at room temperature for 20 minutes. The mixture was then washed thrice with 1 ml of the 1X wash/bind buffer. A final wash was performed using 200 ul of 10 mM Tris-HCl pH 7.5. The beads were then resuspended in 20 ul of nuclease-free water.

The isolated full-length cDNA was then amplified with PCR. The 20 ul of the amplicon-bead conjugate was added to a 30 ul mixture of 10 uM PCR primer, LongAmp Hot Start Taq 2X Master Mix (NEB, M0533S), and nuclease-free water. The PCR primer used was dependent on the flow cell and/or sample used. For the R9 we used cPRM from SQK-PCS111, for the R10 for Sample7 and Sample8 we used cPRM from SQK-PCS111. Amplification in a thermal cycler was performed using the following condition: 94C for 3 minutes, four cycles of 94C for 15 seconds, 56C for 15 seconds, and 65C for 6 minutes, a final extension of 65C for 10 minutes, and a hold of 4C. Clean up was performed using AMPure XP beads (Beckman Coulter^™^ cat # A63881) with a 1.25x solution to beads ratio, and washed twice with freshly made 200 ul of 80% ethanol, and the amplified cDNA was eluted with 15 ul of nuclease-free water.

For the samples processed with a PRM primer, an additional end-prep step needs to be performed before the adapter can be ligated to the full-length transcripts. 200 fmols from the previous step was mixed with the Ultra II End-prep Reaction Buffer and Ultra II End-prep Enzyme Mix (NEB, E7546) and incubated at 20C for 5 minutes and 65C for 5 minutes. Clean up was performed as previously, but with a 1.0X sample-to-beads ratio and eluted with 60 ul of nuclease-free water.

For the R9 and Sample7 and Sample8 R10 samples Rapid Adapter T was added to 35 fmol of cDNA and incubated at room temperature for 5 minutes. For the remaining R10 samples, the end-prepped cDNA was added to a mixture of Ligation Buffer (LNB), NEBNext Quick T4 DNA Ligase, and Ligation Adapter (LA). The mixture was then incubated for 10 minutes at room temperature. Clean up was done with a 2.5x sample-to-beads ratio, washed twice with 250 ul of the Short Fragment Buffer (SFB), and eluded with 25 ul of Elution Buffer.

The Parse samples were processed for Nanopore sequencing using the Ligation Sequencing Kit V14. The libraries were end repaired by mixing the Parse samples with the Ultra II End-prep Reaction Buffer and Ultra II End-prep Enzyme Mix and incubated at 20C for 5 minutes and 65C for 5 minutes. Clean up was performed with a 1.0X sample-to-beads ratio and eluted with 60 ul of nuclease-free water. To add the Nanopore adapter to the cDNA, the end-prepped cDNA was added to a mixture of Ligation Buffer (LNB), NEBNext Quick T4 DNA Ligase, and Ligation Adapter (LA). The mixture was then incubated for 10 minutes at room temperature. Clean up was done with a 2.5x sample-to-beads ratio, washed twice with 250 ul of the Short Fragment Buffer (SFB), and eluded with 25 ul of Elution Buffer.

Samples are loaded into an R9 or R10 PromethION flowcell (FLO-PRO002 or FLO-PRO114M) using the standard Nanopore loading protocol that involves priming the flow cell twice with a flow cell flush and flow cell tether mixture and then adding the Nanopore library that has been mixed the sequencing buffer and loading beads. Libraries were sequenced on a P2-solo and 96 hours.

### Nanopore data processing

Sequencing data was basecalled using Guppy v6.5.7 with super-accuracy (SUP) model. After basecalling, cell UMI barcodes were identified using single cell library vendor supplied tools, including Nanopore’s wf-single-cell pipeline v1.0.1 (https://github.com/epi2me-labs/wf-single-cell) and Parse Bioscience’s pipeline. Since Parse Bioscience’s pipeline does not support transcript-level count matrix. Gene and transcript count matrices are generated using wf-single-cell, SCOTCH, and IsoQuant. To show improved accuracy of R10 flowcells over R9 flowcells, the pipeline was run twice with the R9 and R10 data for sample 7 and sample 8. Once with a barcode edit distance of 1 and a second time with a barcode edit distance of 2. Barcode edit distance is the allowance of a specified number of errors a barcode can have to be classified as a valid cell barcode. To use the Nanopore data on the Parse pipeline, the Nanopore reads would have to be split into artificial paired-end reads. With read 2 containing the Parse barcode sequences and read 1 containing the rest of the reads.

### scRNA-seq pre-processing, cell clustering, and cell type assignment

For PBMC samples, count matrices generated by different computational methods were processed using the Seurat package (v5.0.1)^55, 56^ in R (v4.3.1). Cells with detected genes below 200 or over the 99^th^ percentile or that had mitochondrial gene percentage over 20% were filtered out. The count matrices were then normalized, and the top 2000 variable genes were identified. Top 15 principal components were used to build the neighborhood graph, which was then clustered using the Louvain algorithm with resolution of 0.1. Cell subtyping was performed utilizing singleR and the celldex::DatabaseImmuneCellExpressionData() function^57^. For visual representation and dimensionality reduction, Uniform Manifold Approximation and Projection (UMAP)^58^ was applied.

For raw bam files of cerebral organoid samples, we followed the same preprocessing pipeline and parameters as described in^32^. Count matrices generated by SCOTCH and IsoQuant were processed using the Seurat package. Cells with detected genes below 300 or over 5000 or that had mitochondrial gene percentage over 5% were filtered out. The count matrices were then normalized, and the top 2000 variable genes were identified. Top 30 principal components were used to build the neighborhood graph, which was then clustered using the Louvain algorithm with the resolution that can generate the same number of 22 cell clusters as the original study. To assign cell type labels, we used the AUCell package (v1.22.0)^59^ to calculate cell type-specific enrichment scores for each cell cluster based on marker genes identified in the original study. Cells were then assigned to their respective cell types.

### The SCOTCH pipeline for isoform level characterization of single cell RNA-seq data

The SCOTCH pipeline requires two types of input files: a long-read RNA-seq file provided as a tagged BAM file, and an optional isoform annotation file in GTF format. Upon processing these inputs, SCOTCH generates count matrices at both the gene level and the transcript level, with the capability to identify novel isoforms do not present in the original annotation file. Leveraging these gene and transcript count matrices, SCOTCH enables statistical analyses of the transcriptome across different cell populations, including the detection of genes with differential isoform usage and the identification of isoform switching events. SCOTCH is available at https://github.com/WGLab/SCOTCH. Detailed description of the computational methods is given below.

1. Prepare annotated bam files: Depending on the library type, we utilize either the 'wf-single-cell' or ‘IsoSeq’ pipeline for 10X Genomics libraries or ‘Parse’ for Parse libraries to perform read alignment and barcode identifications. This process produces BAM files annotated with barcode information.
2. Prepare gene annotation files: SCOTCH supports three modes for this step: annotation-only, annotation-free and enhanced-annotation mode. In annotation-only mode, exons are segmented into non-overlapping sub-exons solely based on GTF gene annotation files. In enhanced-annotation mode, read coverage data from BAM files is integrated to refine sub-exon annotations to improve novel isoform identification. This process involves filtering low-coverage regions, identifying splicing positions, and detecting sharp changes in read coverage to discover new exons and more accurately partition existing exons into sub-exons. In annotation-free mode, gene and exon structures are inferred entirely from read coverage data (see Supplementary Material for more detailed information). Detailed gene information, including gene name, strand direction, genome locations, sub-exon coordinates, and transcript annotations, is compiled and stored in a pickle file. Additionally, overlapping genes are grouped into non-overlapping meta-genes for more accurate read-to-gene and read-to-isoform mapping.
3. Assign reads to annotated isoforms: Gene isoforms are treated as specific different combinations of non-overlapping sub-exons. A read is considered aligned to a sub-exon if it covers more than 60% of the sub-exon's length, and is deemed to have skipped the sub-exon if it fails to cover more than 20% of its length, otherwise as ambiguously alignment. Initially, reads are mapped to all annotated isoforms, and any read-isoform pairing is excluded if: (1) the read covers sub-exons not present in the annotated isoform, or (2) the read skips sub-exons that are present in the annotated isoform. Notably, possible read truncations or degradations are not regarded as skipping sub-exons. We specifically implement a dynamic thresholding strategy to handle small-sized sub-exons during read-isoform mapping. Starting from a minimal threshold, we increase it in 10 bp increments until it reaches 80 bp or average sub-exon length of the gene, whichever is smaller. This process continues until we achieve the first unique mapping or, if none is found, the first multiple mapping. Small sub-exons are excluded from decisions on whether a read maps to an isoform to enhance resilience against erroneous mappings and reduce ambiguous alignments. If a read still aligns with multiple annotated isoforms, we select the isoform with the fewest unmapped exons. To further accommodate specific analysis needs, users have the flexibility to adjust parameters of sub-exon coverage and lengths thresholds.
4. Identify novel isoforms: Following previous steps, if a read cannot be mapped to any existing annotated isoforms, it is classified as a ‘novel read’ to be assigned to a novel isoform. To identify novel isoforms, we construct a read-read similarity graph with a maximum of 1500 novel reads at a time. Each node represents a read, and edge weights between nodes are determined by their shared sub-exon mapping patterns. Using the Louvain method, we detect read communities that likely represent the same novel isoforms. For each read community, we determine the novel isoform annotation by calculating the mode of sub-exon mapping encodings across all reads. Once a set of novel isoform candidates is generated, all remaining unmapped reads are realigned to these candidates. Any remaining unmapped reads are grouped into a new batch of 1500, and the process is repeated until no further graphs can be constructed. After generating all novel isoform candidates, novel reads are remapped to the identified isoforms using a relaxed threshold for small sub-exons (the smaller value between 80 bp or the average sub-exon length) to encourage multiple mappings. Redundant isoforms are then eliminated if reads mapped to them can also map to other isoforms, refining the final set of novel isoforms.
5. Handle read ambiguity: Except for cases where a read may map to multiple isoforms—where we reduce ambiguity through dynamic thresholding—reads can potentially map to several genes, either overlapping or non-overlapping (such as chimeric reads due to sequencing artifacts). To resolve these ambiguities, we adopt a mapping score strategy to determine both read-gene and read-isoform assignments. The mapping score is calculated as the percentage of bases aligned to an isoform. Reads are prioritized as follows: first, to genes where the read maps to an existing annotated isoform; second, to genes corresponding to a novel isoform; and lastly, to genes where the read aligns with exons but cannot be classified as either known or novel isoforms.
6. Generate count matrix: During the process of generating the count matrix, if a read maps to potential novel isoform and this mapping is supported by more than 20 reads at the pseudo-bulk level, we classify this as a novel isoform. On the other hand, if fewer than 20 reads support the mapping, it is considered a non-categorized isoform. Utilizing cell barcodes, we can then generate both gene-level and transcript-level count matrices.
7. Transcriptome analysis: SCOTCH can perform transcriptome analysis by testing differential transcript usage on both gene and transcript levels and identify isoform switching events. For gene g with K isoforms, we model the transcript expression level $X_{ck}$ for isoform k in cell c using a Dirichlet-multinomial hierarchical model to account for transcript usage variations among cells. We assume $X_{ck}∼MultiNomial\left(n_{c},\;\pi_{c}\right)$, and $\pi_{c}∼Dirichlet\left(\alpha \right)$. Here, $n_{c}=\sum_{i=1}^{K}X_{k}$ is the total transcript counts for the gene in the cell. $\pi_{c}=\left(\pi_{c,1},\;\ldots ,\;\pi_{c,K}\right)$ is cell-specific transcript usage. $\alpha =\left(\alpha_{1},\;\ldots ,\;\alpha_{K}\right)$ are the concentration parameters of the Dirichlet distribution we will estimate, and the average transcript usage for the cell population is $\bar{\pi }=\left({\bar{\pi }}_{1},\;\ldots ,\;{\bar{\pi }}_{K}\right)$ with ${\bar{\pi }}_{k}=\frac{\alpha_{k}}{\sum_{i=1}^{K}\alpha_{i}}$ Similar with LongCell, we define $\phi =\frac{1}{1+\sum_{i=1}^{K}\alpha_{i}}$ as a mean-invariant over-dispersion parameter, representing inter-cell heterogeneity. A small ϕ suggests that cells are likely to express various isoforms with similar usage proportions across the cell population, and a large ϕ value indicates a more exclusive expression manner, with each cell predominantly expressing one isoform while different cells may express others. Our goal is to estimate $\bar{\pi }$ using maximum likelihood estimation and compare between different cell populations A and B using likelihood ratio test. On the gene level, we test $H_{0}:{\bar{\pi }}_{k}^{A}={\bar{\pi }}_{k}^{B}$ for k=1, …, K vs $H_{1}:{\bar{\pi }}_{k}^{A}\neq {\bar{\pi }}_{k}^{B}$ for any 1≤k≤K. On the transcript level, we test $H_{0}:{\bar{\pi }}_{k}^{A}={\bar{\pi }}_{k}^{B}$ vs $H_{1}:{\bar{\pi }}_{k}^{A}\neq {\bar{\pi }}_{k}^{B}$ for isoform k. Note that, we aggregate rare isoforms into one isoform type if $\sum_{c}X_{ck}\leq 0.05\sum_{c}n_{c}$, for 1≤k≤K, and only analyze genes with over 20 reads mapped to any known or novel isoforms. We define isoform switching events if the dominant isoform except the aggregated rare isoforms is different between two cell populations inferred by ${\bar{\pi }}^{A}$ and ${\bar{\pi }}^{B}$. The effect size is defined as $\left|\left({\bar{\pi }}_{a}^{A}-{\bar{\pi }}_{b}^{B}\right)+\left({\bar{\pi }}_{b}^{B}-{\bar{\pi }}_{b}^{A}\right)\right|$ where isoform a and b are dominant isoforms for cell populations A and B, respectively.

### Simulation study

We simulated ground-truth nanopore scRNA-seq data for two distinct cell populations using a modified version of lrgasp-simulation (https://github.com/andrewprzh/lrgasp-simulation/tree/main). While originally developed for bulk long-read RNA-seq data, we adapted the simulation tool for single-cell applications by introducing between-cell transcript usage variations. We used GENCODE annotations for all 1080 genes that have at least two known isoforms on chromosome 6. To simulate the discovery of novel isoforms, we randomly removed 2466 (30%) isoforms and designated them as ground-truth novel isoforms. The remaining 5762 (70%) isoforms, covering all 1080 genes, were used as the existing annotation file for input across all tools. For a given gene g, let K represent the true number of isoforms based on the reference annotation file. We sampled gene expression counts $n_{c}$ for the cell c from the distribution $n_{c}\sim \mathrm{Pois}\left(\lambda_{c}\right)$, where $\lambda_{c}\sim U\left(0,\;15\right)$. We then sampled the true transcript usage for the cell from the distribution $\pi_{c}\sim \mathrm{DIR}\left(\alpha \right)$, where each $\alpha_{k}$ is drawn from a $\mathrm{Gamma}\left(2,\;2\right)$ distribution. The expected transcript counts for cell c are $x_{c}=\left(x_{\mathrm{c,}1},\;x_{\mathrm{c,}2},\;\cdots ,\;x_{\mathrm{c,K}}\right)$, where $x_{\mathrm{c,K}}=n_{c}\pi_{c}$. Based on transcript lengths, transcript per million (TPM) were calculated as input for the lrgasp-simulation. To control gene counts and underlying ground truth isoform usage, we simulated reads for each cell expressing one gene independently. We simulated 1000 cells for each of the two populations, with 540 genes sharing the same a values and the remaining 540 having different a values to simulate differential transcript usage (DTU) between populations. In total, 15,381,985 reads were simulated across the two populations. The simulated FASTA files were combined and aligned to the human genome using minimap2. Cell barcodes and UMIs were added as tags in the BAM files based on the cell origins of the reads.

## Supplementary Material

Supplement 1

## Figures and Tables

**Table 1.** Summary of human PBMC benchmarking datasets generated in the current study

Sample Name	Sequencing Platform	Flowcell	Single Cell Library
Sample 7	Illumina		10x Genomics 3' v3
Sample 7	Illumina		Parse Biosciences
Sample 7	Nanopore	R9	10x Genomics 3' v3
Sample 7	Nanopore	R10	10x Genomics 3' v3
Sample 7	Nanopore	R10	Parse Biosciences
Sample 8	Illumina		10x Genomics 3' v3
Sample 8	Illumina		Parse Biosciences
Sample 8	Nanopore	R9	10x Genomics 3' v3
Sample 8	Nanopore	R10	10x Genomics 3' v3
Sample 8	Nanopore	R10	Parse Biosciences

**Table 2.** Number of genes with significant differential transcript usage (DTU)

		B cells	Monocytes	NK cells	CD4+ T cells	CD8+ T cells
10X - ont - SCOTCH	Sample 7	2420	3590	549	519	190
	Sample 8	3134	4736	1436	653	144
	Consensus	1835	2990	448	315	81

10X - ont - SCOTCH.ao	Sample 7	2797	4283	731	678	221
	Sample 8	3501	5414	1674	698	152
	Consensus	2136	3693	565	364	89

10X - ont – wf-single-cell	Sample 7	1605	2443	423	313	68
	Sample 8	1881	2960	953	267	52
	Consensus	862	1577	241	123	13

10X - ont - IsoQuant	Sample 7	209	447	35	41	23
	Sample 8	317	618	103	52	12
	Consensus	143	334	23	24	6

Parse - ont - SCOTCH	Sample 7	142	273	19	28	1
	Sample 8	27	73	8	1	2
	Consensus	16	43	3	0	0

All DTU genes were identified with a p-adj ≤ 0.01 determined by the False Discovery Rate (FDR) method. Data are generated by 10X or Parse libraries and preprocessed by different computational pipelines and are denoted by single cell libraries – sequencing technology - preprocessing pipeline.

## Data Availability

Raw data of two PBMC samples using both 10X Genomics and Parse libraries, Illumina and nanopore sequencings have been deposited to Sequence Read Archive (SRA) at the National Center for Biotechnology Information (NCBI) under accession number PRJNA1105904.
