# Supplementary material for: Single-Cell Omics for Transcriptome CHaracterization (SCOTCH): isoform-level characterization of gene expression through long-read single-cell RNA sequencing
Source: bioRxiv. 2025 Feb 6:2024.04.29.590597. Originally published 2024 Apr 30. Preprint. [Version 3] doi: 10.1101/2024.04.29.590597 (PMC11092450; doi:10.1101/2024.04.29.590597)
Supplement: Supplement 1 [file media-1.pdf]

Supplementary Materials

Supplementary tables and figures

Table S1. Summary statistics of the single cell data sets on PBMC. For some measures on Nanopore R9 flowcells, the statistic for using edit distance of 1 or 2 (ED1/ED2) are listed. For Illumina data and Nanopore R10 data, only ED1 was used.

Single Cell Processing Method	Sequencing Platform (Sample ID)	Sequence Saturation (ED1/ED2)	UMI Count Threshold (ED1/ED2)	Mean Reads Per Cell (ED1/ED2)	Median Reads Per Cell (ED1/ED2)	Median Genes Per Cell (ED1/ED2)	Median Transcripts Per Cell (ED1/ED2)	Median UMI Count Per Cell (ED1/ED2)
10X	Illumina (Sample 7)	71.7%	500	51,542	---	2,723	---	8,376
10X	ONT-R9 (Sample 7)	34.7% / 35.8%	507 / 565	11,983 / 13,570	10,859 / 12,347	2,279 / 2,398	1,280 / 1,360	6,954 / 7,724
10X	ONT-R10 (Sample 7)	46.0% / 46.6%	597 / 625	16,350 / 17,375	14,890 / 15,813	2,520 / 2,581	1,426 / 1,467	7,761 / 8,203
10X	Illumina (Sample 8)	65.7%	500	37,145	---	2,597	---	6,893
10X	ONT-R9 (Sample 8)	28.6% / 29.5%	531 / 579	8,760 / 9,866	7,894 / 8,876	2,060 / 2,177	1,097 / 1,173	5,493 / 6,096
10X	ONT-R10 (Sample 8)	43.9% / 44.4%	587 / 631	14,398 / 15,219	12,954 / 13,711	2,471 / 2,528	1,359 / 1,397	6,967 / 7,320
Parse	Illumina (Sample 7)	62.3%	---	70,992	---	2,338	5,078	---
Parse	ONT-R10 (Sample 7)	20.7%	---	7,129	---	1,116	1,758	---
Parse	Illumina (Sample 8)	62.0%	---	32,903	---	1,407	2,282	---
Parse	ONT-R10 (Sample 8)	20.0%	---	3,144	---	563	742	---

Table S2. Isoform categories identified by SCOTCH for Nanopore_R10 data.

	Sample7<br>n (%)	Sample8<br>n (%)
**Known Isoforms**	9339660 (34.33)	11020357 (37.26)
**Novel Isoforms**	5066021 (18.65)	5985206 (20.21)
**Uncategorized**	10659519 (47.02)	15093657 (42.53)
**Total Reads**	32099220	25065200

Figure S1. Sashimi plot for the *IL27RA* gene. SCOTCH pipeline identifies known (ENST00000263379) and novel (Novel-Isoform-3) isoforms for the *IL27RA* gene across two samples. The novel isoform is categorized by including only the first two exons at the 5' end. This annotation of the novel isoform also indicates the limitation of SCOTCH in detecting novel exons, such as intron retention. Results shown are based on 10X-ONT PBMC data of sample 8.

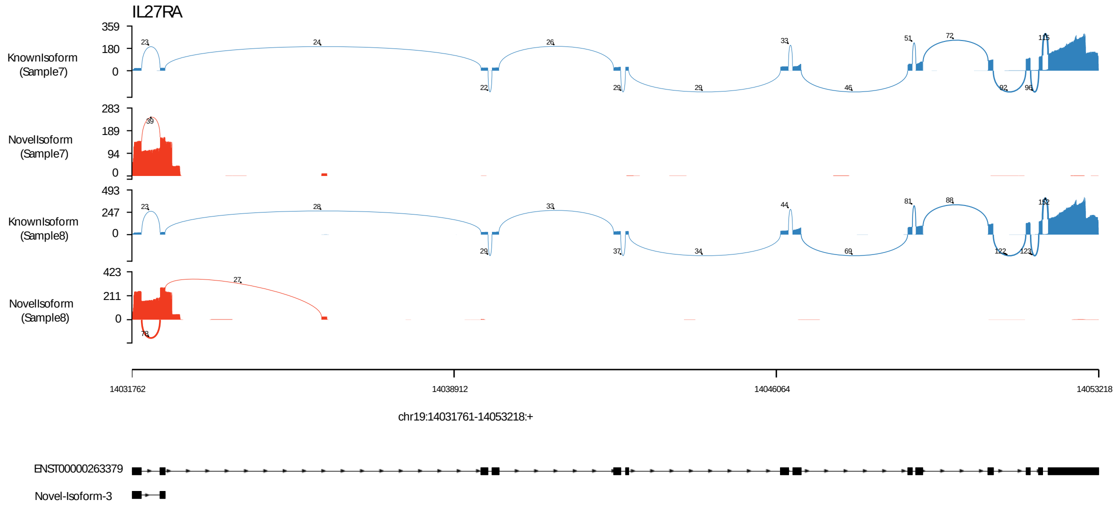
IL27RA
KnownIsoform (Sample7)
NovelIsoform (Sample7)
KnownIsoform (Sample8)
NovelIsoform (Sample8)
14031762
14038912
14046064
14053218
chr19:14031761-14053218:+
ENST00000263379
Novel-Isoform-3
